# KIFC1 depends on TRIM37-mediated ubiquitination of PLK4 to promote centrosome amplification in endometrial cancer

**DOI:** 10.1038/s41420-024-02190-1

**Published:** 2024-09-30

**Authors:** Kening Zhou, Yingying He, Xi Lin, Huihao Zhou, Xiaomin Xu, Jingui Xu

**Affiliations:** 1 Department of Gynaecology, The Quzhou Affiliated Hospital of Wenzhou Medical University, Quzhou People’s Hospital, Quzhou City, Zhejiang Province 324000 China; 2 Department of Pathology, The Quzhou Affiliated Hospital of Wenzhou Medical University, Quzhou People’s Hospital, Quzhou City, Zhejiang Province 324000 China

**Keywords:** Cancer, Biomarkers

## Abstract

Endometrial cancer (EC), as one of the most common cancers, severely threatens female reproductive health. Our previous study has shown that Kinesin family member C1 (KIFC1) played crucial roles in the progression of EC. In addition, abnormal centrosome amplification, which was reported to be partially regulated by KIFC1, usually occurred in different cancers. However, whether KIFC1 promoted EC through centrosome amplification and the potential mechanism remain to be revealed. The present study demonstrated that overexpressed KIFC1, which exhibited a worse prognosis, had a positive correlation with an increased number of centrosomes in human EC samples. In addition, KIFC1 overexpression in EC cells prompted centrosome amplification, chromosomal instability, and cell cycle progression. Moreover, we demonstrated that KIFC1 inhibited E3 ubiquitin-protein ligase TRIM37 to maintain the stability of PLK4 by reducing its ubiquitination degradation, and finally promoting centrosome amplification and EC progression in vitro. Finally, the contributing role of KIFC1 and the inhibitory effect of TRIM37 on EC development and metastasis was verified in a nude mouse xenograft model. Our study elucidated that KIFC1 depends on TRIM37-mediated reduced ubiquitination degradation of PLK4 to promote centrosome amplification and EC progression, thus providing a potential prognostic marker and promising therapeutic target for EC in the future.

## Introduction

Endometrial cancer (EC) is one of the most prevalent cancers in women all over the world [1]. Although approximately 75% of EC were diagnosed at an early stage, and the survival rate was 75%, its average lifetime risk is almost 3% [2]. These patients with low-grade and early-stage EC exhibited pretty prognoses. In addition, a previous study has reported that only 15% of patients were diagnosed before 50 years old, and only 5% before 40 years old [1]. Thus, even with great improvements in diagnostic and therapeutic interventions in recent years, the outcomes of those patients with high-grade and metastatic or recurrent cancer still remain poor. Therefore, further investigation of the tumorigenic mechanism and finding the potential effective biomarkers are urgently required to improve the prognosis for EC.

Kinesin family member C1 (KIFC1), a nonessential minus end-directed motor of the kinesin-14 family, is an essential centrosome clustering molecule [3]. Higher expression of KIFC1 was observed in several types of cancers and correlated with poor prognosis [4–7]. Thus, KIFC1 was regarded as a promising chemotherapy target for cancer treatment. Previous studies have reported that KIFC1 participated in various physiological processes, such as the maintenance of spindle poles in mitosis [8], actin dynamics in oocyte meiosis [9], nutritional metabolism [7], DNA transport [10], spindle assembly and chromosome segregation [8]. Due to the pivotal role of KIFC1 in regulating spindle assembly and centrosome clustering, which further resulted in genomic instability, whether KIFC1 promotes the EC progression dependent on centrosome still needs investigation.

In animals, more than two centrosomes at the onset of mitosis usually contribute to chromosomal instability and subsequent different tumorigenesis. Under these circumstances, normal cells can undergo repair or apoptosis to solve this problem, whereas cancer cells gradually learn to cope with this matter by a unique mechanism, known as centrosome clustering [11]. Previous studies have shown that centrosome amplification widely occurred in various cancers, such as gastric cancer (GC)[12], prostate cancer (PCa) [4], ovarian cancer (OC) [3], and breast cancer [5]. During the process of mitosis, centrosomes are responsible for catalyzing microtubule generation for spindle assembly, while centrosomes only duplicate once per cell cycle which was controlled by polo-like kinase 4 (PLK4) [13]. Therefore, PLK4 played an important role in centrosome replication. One study has indicated that PLK4-related centrosome amplification was involved in the GC progression [12]. However, the role of PLK4-controlled centrosome amplification in KIFC1 regulating EC progression remains unknown.

In this study, we first analyzed the KIFC1 expression and its relationship with the overall survival of EC patients based on the database. In addition, the protein expressions of KIFC1 and γ-tubulin, the marker of centrosome, in EC specimens were analyzed and further proved by IHC staining. Thereafter, the regulatory effect of KIFC1 on centrosome amplification, cell cycle progression, and chromosomal instability was evaluated based on EC cell lines and the potential mechanism of KIFC1 affecting centrosome amplification. The results based on the above experiments inferred that KIFC1 promoted centrosome amplification and EC progression dependent on PLK4, which also contributed to cell survival, proliferation, invasion, and migration. Co-IP assays indicated that KIFC1 did not interact with PLK4, whereas TRIM37 interacted with PLK4, and KIFC1 interacted with TRIM37. Mechanically, KIFC1 decreased the expression of TRIM37 to reduce the PLK4 ubiquitination degradation and enhance its stability which finally led to EC progression. Furthermore, the contributing effect of KIFC1 and the inhibitory effect of TRIM37 on EC development and metastasis were verified in a mouse model. Our study showed great significance in providing new therapeutic targets for EC patients.

## Results

### Higher expression of KIFC1 and more centrosome amplification were observed in EC

**Fig. 1:**
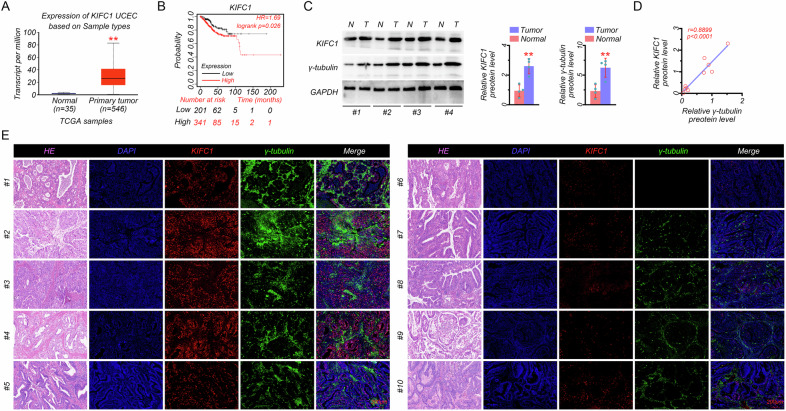
Higher expression of KIFC1 and more centrosome amplification were observed in EC. **A** Gene expression of KIFC1 in normal (*n* = 35) and EC samples (*n* = 546) based on the TCGA database. **B** Survival curves of KIFC1 expression in EC patients from Kaplan–Meier plotter. **C** The protein expressions of KIFC1 and γ-tubulin in EC specimens (T) and adjacent normal tissues (N). **D** The correlation analysis between KIFC1 and γ-tubulin protein expressions based on EC specimens and normal tissues. **E** The H & E staining and immunofluorescence analysis with KIFC1 and γ-tubulin antibodies in EC specimens (left) and adjacent normal tissues (right) (scale bar: 200 μm). **P < 0.01.

We first evaluated the expression pattern of KIFC1 in human EC. Results from the TCGA database showed that the mRNA expression of KIFC1 was upregulated in primary tumors compared with the normal tissues (Fig. 1A). Kaplan–Meier curves of OS in EC patients from the Kaplan–Meier plotter showed that patients with higher KIFC1 expression exhibited worse prognosis (Fig. 1B). Due to the role of KIFC1 as a centrosome clustering molecule, we further detected the protein expressions of KIFC1 and γ-tubulin, the marker of centrosome, in EC specimens. Our results indicated that KIFC1 and γ-tubulin were significantly overexpressed in EC tissues (Fig. 1C), and a strong positive correlation was observed between the protein expressions of KIFC1 and γ-tubulin (Fig. 1D). In addition, H & E staining showed no obvious inflammatory infiltration and injury between tumor and normal tissues (Fig. 1F). However, IF staining with KIFC1 and γ-tubulin antibodies (Fig. 1F and S1A) further proved that the relative fluorescence intensity of KIFC1 along with γ-tubulin was significantly higher in the tumor tissues (samples 1–5) than that in the normal tissues (samples 6–10), indicating an increased number of centrosomes. Together, these data suggested that KIFC1, strongly associated with a poor prognosis, was highly expressed in EC, and abnormal centrosome amplification was also increased during the process.

### KIFC1 promoted centrosome amplification

**Fig. 2:**
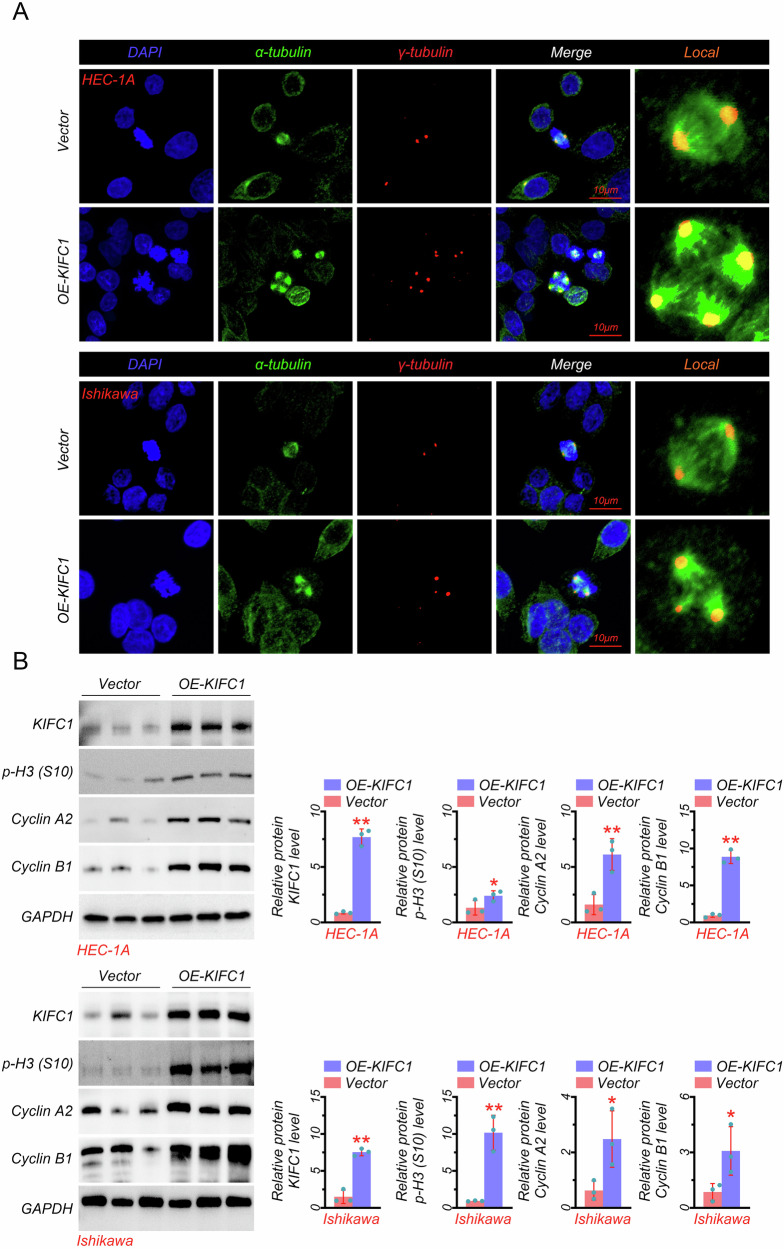
KIFC1 promoted centrosome amplification. **A** The immunofluorescence analysis with γ-tubulin and α-tubulin antibodies in KIFC1-overexpressed EC cells (scale bar: 10 μm). **B** The protein expressions in KIFC1-overexpressed EC cells. *P < 0.05, **P < 0.01.

We further evaluated the correlation between KIFC1 expression and centrosome amplification based on EC cell lines. Results showed that KIFC1 overexpression increased the accumulation of γ-tubulin and α-tubulin in HEC-1A cells (Fig. 2A), which was further proved in Ishikawa cells (Fig. 2A). More importantly, γ-tubulin was translocated into the nucleus in KIFC1-overexpressed EC cells (Fig. 2A), indicating that the abnormal centrosome numbers were induced by KIFC1. Statistical analysis of the centrosome amplification showed that the presence of centrosome amplification was quite higher in the OE-KIFC1 group (~22%) than in the Vector group (10%), whether in HEC-1A or Ishikawa cells (Fig. S1B). Besides, disturbance of centrosome amplification can further damage the cell cycle and induce chromosomal instability, thus we also detected the related proteins expression. Results in Fig. 2B indicated that the protein expressions of KIFC1, p-H3, Cyclin A2, and Cyclin B1 were markedly increased in KIFC1-overexpressed HEC-1A cells, which was also observed in KIFC1-overexpressed Ishikawa cells. Moreover, KIFC1 overexpression in HEC-1A and Ishikawa cells significantly contributed to the increased mRNA expressions of cyclin A2 and cyclin B1 (Fig. S1C). In addition, we also measured the protein expression of the corresponding kinase (CDK1 and CDC2) in this phase of the cycle. Results in Fig. S1D showed that KIFC1 overexpression in HEC-1A and Ishikawa cells also markedly increased the protein levels of CDK1 and CDC2. Together, KIFC1 promoted centrosome amplification, which also induced cell cycle progression and chromosomal instability.

### KIFC1 promoted centrosome amplification dependent on PLK4

**Fig. 3:**
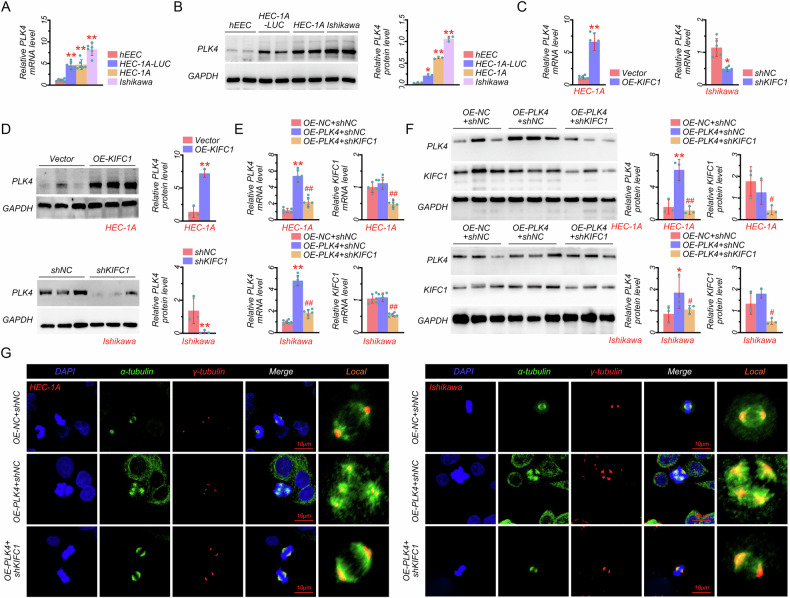
KIFC1 promoted centrosome amplification dependent on PLK4. **A** The mRNA expression of PLK4 in EC cell lines. **B** The protein expression of PLK4 in EC cell lines. **C** The mRNA expression of PLK4 in KIFC1-overexpressed HEC-1A and KIFC1-knockdown Ishikawa cells. **D** The protein expression of PLK4 in KIFC1-overexpressed HEC-1A and KIFC1-knockdown Ishikawa cells. **E** The mRNA expressions of PLK4 and KIFC1 in PLK4-overexpressed and KIFC1-knockdown EC cell lines (HEC-1A and Ishikawa). **F** The protein expressions of PLK4 and KIFC1 in PLK4-overexpressed and KIFC1-knockdown EC cell lines (HEC-1A and Ishikawa). **G** The immunofluorescence analysis with γ-tubulin and α-tubulin antibodies in PLK4-overexpressed and KIFC1-knockdown EC cell lines (HEC-1A and Ishikawa) (scale bar: 10 μm). *P < 0.05, **P < 0.01, #*P* < 0.05, ##*P* < 0.01.

Previous studies have shown that PLK4 played an essential role in centrosome amplification [12]; whether PLK4 was involved in KIFC1 regulating centrosome amplification was still unknown. Thus, we further evaluated the role of PLK4 in EC cells. Results in Fig. 3A and B showed that the mRNA and the protein level of PLK4 were significantly improved in EC tumor cell lines (HEC-1A-LUC, HEC-1A, and Ishikawa) compared with the normal hEEC. Besides, we evaluated the effect of KIFC1 on PLK4. Results showed that the gene expression of PLK4 was markedly increased in the KIFC1-overexpressed HEC-1A cells compared with the Vector cells, which was decreased in the KIFC1-knockdown Ishikawa cells compared with the shNC cells (Fig. 3C). Similarly, the protein expression of PLK4 was significantly increased in the KIFC1-overexpressed HEC-1A cells and markedly reduced in the KIFC1-knockdown Ishikawa cells (Fig. 3D). These data suggested that KIFC1 might contribute to the expression of PLK4 in EC cell lines. To further prove the hypothesis, PLK4 overexpressing plasmid was constructed and transfected into EC cells. Our data showed that the gene and protein levels of PLK4 were markedly higher in HEC-1A cells transfected with OE-PLK4 and shNC plasmids compared with cells transfected with OE-NC and shNC plasmids (Fig. 3E and F), whereas PLK4 overexpression did not affect KIFC1 expression (Fig. 3E and F). Besides, the mRNA and protein levels of PLK4 and KIFC1 were pronouncedly dropped in the HEC-1A cells transfected with OE-PLK4 and shKIFC1 plasmids compared with cells transfected with OE-PLK4 and shNC plasmids (Fig. 3E and F). More importantly, similar pattern results were also observed in Ishikawa cells transfected with OE-PLK4 and shKIFC1 plasmids (Fig. 3E and F). Additionally, the Pearson correlation analysis in EC patients demonstrated a strong positive correlation between the mRNA expression of KIFC1 and PLK4 (Fig. S1H). The above results confirmed that KIFC1 promoted the expression of PLK4. Furthermore, we measured the effect of PLK4 overexpression and KIFC1 silencing on centrosome amplification. Images of IF staining showed that PLK4 overexpression significantly promoted the enrichment of γ-tubulin and α-tubulin, markedly inhibited by KIFC1 silencing in HEC-1A and Ishikawa cells (Fig. 3G). The percent of centrosome amplification was also increased in the PLK4-overexpressed HEC-1A (~35%), and Ishikawa cells (~25%) compared to the OE-NC + shNC group (10%), which was abolished by KIFC1 knockdown (Fig. S1E). Together, these results demonstrated that PLK4 participated in the process of KIFC1-promoting abnormal centrosome amplification.

### PLK4 promoted the EC progression

**Fig. 4:**
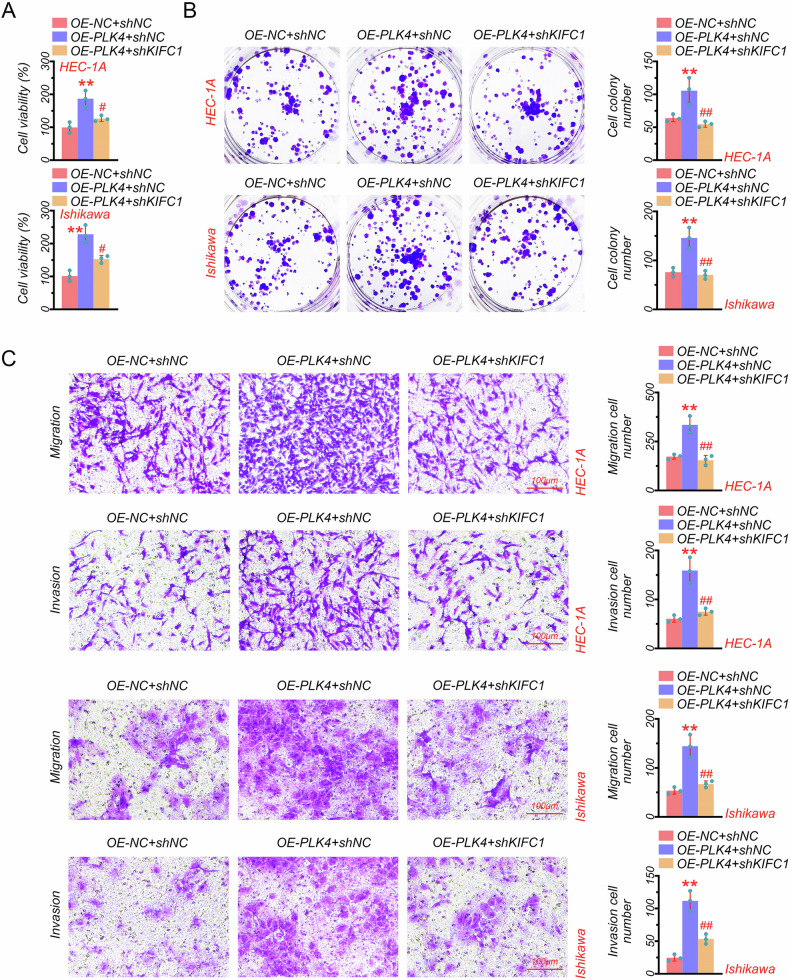
PLK4 promoted the EC progression. **A** CCK8 assay in PLK4-overexpressed and KIFC1-knockdown EC cell lines (HEC-1A and Ishikawa). **B** Colony formation assay in PLK4-overexpressed and KIFC1-knockdown EC cell lines (HEC-1A and Ishikawa). **C** Transwell assay and quantitative analysis of migratory and invasive abilities of PLK4-overexpressed and KIFC1-knockdown EC cell lines (HEC-1A and Ishikawa) (scale bar: 100 μm). **P < 0.01, ##*P* < 0.01.

Based on our previous results that KIFC1 contributed to cell proliferation, migration, and invasion in EC cells, as well as the involvement of PLK4 on KIFC1 regulating centrosome amplification in EC patients and cells, we further evaluated the effect of PLK4 on EC progression. Results showed that PLK4 overexpression significantly increased cell viability and promoted cell proliferation, migration, and invasion in HEC-1A cells, which was reversed by KIFC1 knockdown (Fig. 4A–C). Furthermore, similar results were also observed in Ishikawa cells transfected with OE-PLK4 and shKIFC1 plasmids (Fig. 4A–C). Together, PLK4 markedly contributed to the EC progression.

### KIFC1 controlled the ubiquitination of PLK4

**Fig. 5:**
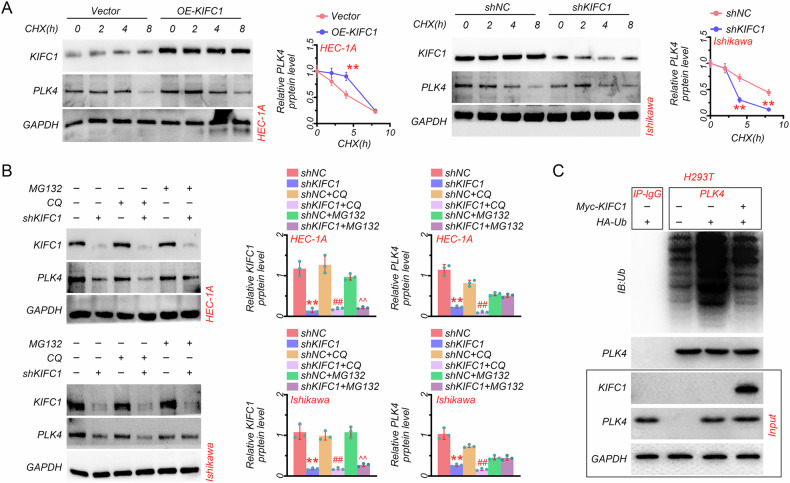
KIFC1 controlled the ubiquitination of PLK4. **A** CHX chase analysis of PLK4 protein half-life after KIFC1 overexpression and knockdown in EC cell lines (HEC-1A and Ishikawa). **B** The protein expression of KIFC1 and PLK4 in HEC-1A and Ishikawa cells treated with MG132 or CQ after KIFC1 knockdown. **C** Results of the ubiquitination level of PLK4 in HEK293T cells after KIFC1 overexpression detected by ubiquitination assay. **P < 0.01, ##*P* < 0.01, ^^*P* < 0.01.

To illustrate the mechanism of KIFC1 regulating PLK4 expression, KIFC1 overexpressing and silencing cells were treated with cycloheximide (CHX), a protein synthesis inhibitor. Results indicated that PLK4 degradation was lower in KIFC overexpressing HEC-1A cells than in Vector cells, and PLK4 degradation was faster in KIFC silencing Ishikawa cells than in shNC cells (Fig. 5A). These data further demonstrated that KIFC1 overexpression increased the stability of PLK4. The autophagy-lysosome and ubiquitin-proteasome pathways are the primary protein degradation pathways. Thus, EC cells were treated with the ubiquitin-proteasome pathway inhibitor MG132 and autophagy-lysosome pathway inhibitor chloroquine diphosphate (CQ). Our results showed that KIFC1 knockdown indeed reduced the protein levels of KIFC1 and PLK4 in HEC-1A and Ishikawa cells (Fig. 5B) as before. In addition, CQ treatment showed no impact on the protein level of PLK4 in KIFC1-knockdown HEC-1A and Ishikawa cells, whereas MG132 could reverse the decreased protein level of PLK4 by KIFC1 knockdown (Fig. 5B), inferring that KIFC1 regulated the stability of PLK4 mainly through the ubiquitination pathway. More importantly, in vitro ubiquitination assay also showed that KIFC1 overexpression significantly inhibited PLK4 ubiquitination in HEK293T cells (Fig. 5C). Thus, these findings further proved that KIFC1 enhanced the stability of PLK4 by inhibiting its ubiquitination.

### KIFC1 regulated the EC progression by controlling TRIM37 expression

**Fig. 6:**
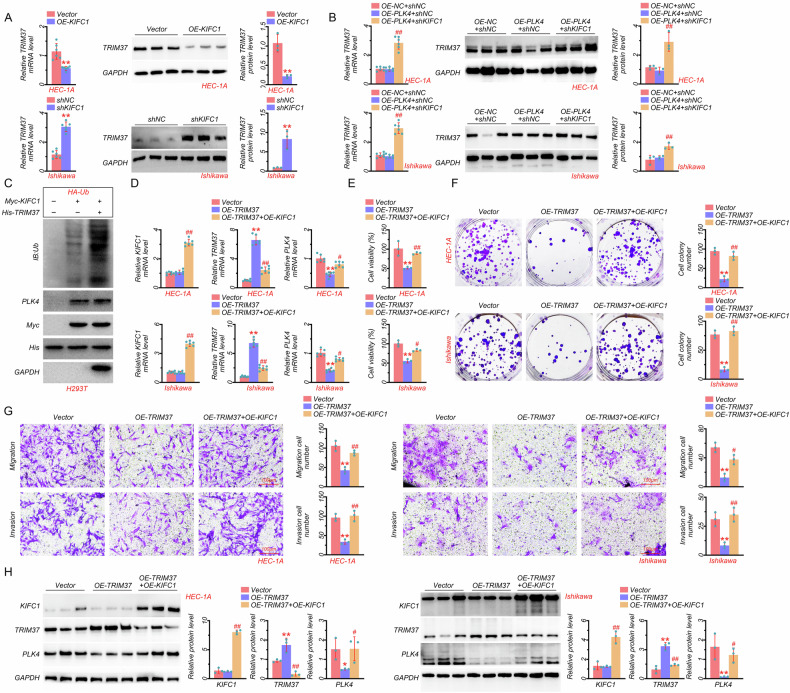
KIFC1 regulated the EC progression by controlling TRIM37 expression. **A** The mRNA and protein expressions of TRIM37 in KIFC1-overexpressed HEC-1A and KIFC1-knockdown Ishikawa cells. **B** The mRNA and protein expressions of TRIM37 in PLK4-overexpressed and KIFC1-knockdown EC cell lines (HEC-1A and Ishikawa). **C** Results of the ubiquitination level of PLK4 in HEK293T cells after KIFC1 or TRIM37 overexpression detected by ubiquitination assay. **D** The mRNA expressions of KIFC1, TRIM37, and PLK4 in TRIM37- and KIFC1-overexpressed EC cell lines (HEC-1A and Ishikawa). **E** CCK8 assay in TRIM37- and KIFC1-overexpressed EC cell lines (HEC-1A and Ishikawa). **F** Colony formation assay in TRIM37- and KIFC1-overexpressed EC cell lines (HEC-1A and Ishikawa). **G** Transwell assay and quantitative analysis of migratory and invasive abilities of TRIM37- and KIFC1-overexpressed EC cell lines (HEC-1A and Ishikawa) (scale bar: 100 μm). **H** The protein expressions of KIFC1, TRIM37, and PLK4 in TRIM37- and KIFC1-overexpressed EC cell lines (HEC-1A and Ishikawa). *P < 0.05, **P < 0.01, #*P* < 0.05, ##*P* < 0.01.

To investigate the potential mechanism of KIFC1 regulating PLK4 degradation, a Co-IP assay was conducted, and results showed that KIFC1 did not interact with PLK4 directly (Fig. S1F). We further hypothesized that some ubiquitinating-related enzymes may be involved in the process. TRIM family members were reported to regulate the oncogenesis, development, and progression of various human cancers [14]. Among these, TRIM37, as the E3 ubiquitin-protein ligase, can target PLK4 participating in the formation of acentrosomal spindle assembly [13]. Therefore, we first evaluated the impact of KIFC1 on TRIM37 expression in EC cell lines, results showed that KIFC1 overexpression in HEC-1A cells significantly inhibited the mRNA and protein expression of TRIM37, which was reversed in KIFC1-knockdown Ishikawa cells (Fig. 6A). Moreover, we found that PLK4 overexpression showed no impact on the gene and protein levels of TRIM37, which was markedly increased in PLK4-overexpressed and KIFC1-knockdown HEC-1A cells compared with that in PLK4-overexpressed cells (Fig. 6B). Subsequently, we also observed the similar results in PLK4-overexpressed and KIFC1-knockdown Ishikawa cells (Fig. 6B). These data suggested that TRIM37 was the potential downstream target of KIFC1, and can target PLK4. In addition, Co-IP assays showed that PLK4 interacted with TRIM37 directly (Fig. S1F). Furthermore, we evaluated the effect of TRIM37 on PLK4 ubiquitination. Results showed that TRIM37 overexpression significantly increased PLK4 ubiquitination (Fig. 6C), and KIFC1 interacted with TRIM37 directly based on Co-IP assay (Fig. S1G), which together indicated that KIFC1 reduced TRIM37 expression to inhibit the direct binding of TRIM37 and PLK4, thereby reducing PLK4 ubiquitination and enhancing its stability. We then used TRIM37 overexpression plasmid in HEC-1A and Ishikawa cells to detect the effect of TRIM37 on KIFC1 and PLK4 levels. As shown in Fig. 6D and H, TRIM37 overexpression markedly reduced the mRNA and protein levels of PLK4, with no impact on KIFC1 expression. Conversely, KIFC1 overexpression attenuated the downregulation of PLK4 induced by TRIM37 (Fig. 6D and H), further indicating that KIFC1 controlled TRIM37 expression to enhance PLK4 stability. In addition, the mRNA expression of TRIM37 was strongly negatively correlated with the mRNA expressions of KIFC1 and PLK4 based on EC samples (Fig. S1G). Besides, the effect of TRIM37 on EC progression was also measured. Our data exhibited that TRIM37 overexpression pronouncedly inhibited cell survival, proliferation, migration, and invasion in HEC-1A and Ishikawa cells, which was reversed by KIFC1 overexpression (Fig. 6E–G), indicating the inhibitory effect of TRIM37 on the development of EC. Together, these results suggested that KIFC1 downregulated the TRIM37 expression to enhance the stability of PLK4, and further promoted the EC progression.

### KIFC1 depended on TRIM37 to promote tumor metastasis in vivo

**Fig. 7:**
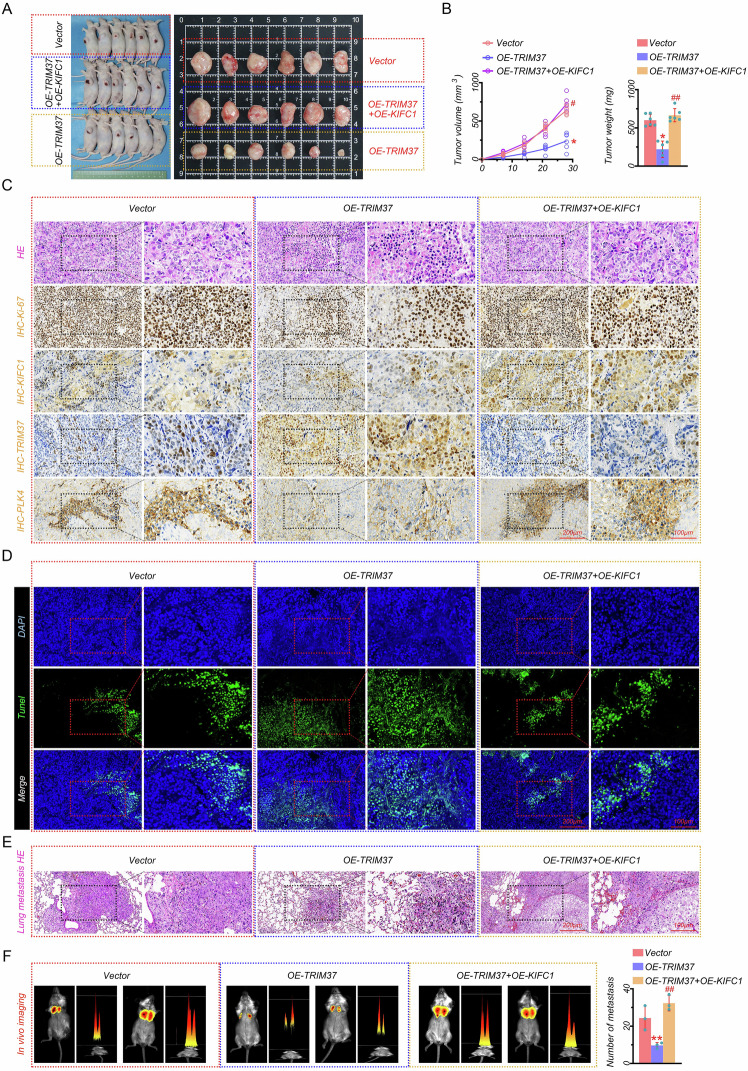
KIFC1 promoted tumor metastasis dependent on TRIM37 in vivo. **A** General images of mice and tumors in the subcutaneous xenograft of HEC-1A cells after TRIM37 and KIFC1 overexpression. **B** The tumor volume and weight. **C** Representative H & E staining and IHC staining of Ki-67, KIFC1, TRIM37, and PLK4 in tumors (scale bar: 200 μm for left; 100 μm for right). **D** Representative TUNEL images of tumors (scale bar: 200 μm for left; 100 μm for right). **E** Representative H & E staining images of lung tissues (scale bar: 200 μm for left; 100 μm for right); **F** Fluorescence images (left) and quantitative analysis (right) of mouse lung metastasis. *P < 0.05, **P < 0.01, #*P* < 0.05, ##*P* < 0.01.

To confirm the effect of KIFC1 and TRIM37 on EC metastasis in vivo, we performed a mouse xenograft assay and lung metastasis assay. Results showed that TRIM37 overexpression markedly inhibited tumor growth, which was abolished by KIFC1 overexpression (Fig. 7A and B). Moreover, TRIM37 overexpression attenuated inflammatory injury of tumors as accessed by H & E staining, as well as decreased proliferation measured by Ki-67 staining (Fig. 7C). Similar to the results of EC cells, TRIM37 overexpression reduced the content of PLK4, increased the level of TRIM37, and had no impact on KIFC1 in mouse model (Fig. 7C). Besides, TRIM37 and KIFC1 overexpression contributed to the inflammatory infiltration of tumors, promoted proliferation, increased the contents of KIFC1 and PLK4, but reduced the level of TRIM37 (Fig. 7C). In addition, TUNEL staining further indicated that TRIM37 resulted in increased apoptosis to suppress tumor growth, which was inhibited by KIFC1 overexpression (Fig. 7D). More importantly, the lung metastasis was evaluated by H & E staining of lung tissues and fluorescence images of mice. Our data showed that TRIM37 overexpression inhibited inflammatory injury and tumor metastasis in lung tissues, which was reversed by KIFC1 overexpression (Figs. 7E and F). In conclusion, these results demonstrated the contributing role of KIFC1 and the inhibitory effect of TRIM37 in the proliferation and metastasis of EC in vivo.

## Discussion

EC is the seventh most prevalent cancers for women all over the world [15]. Although a good prognosis is observed in patients with low-grade and early-stage, there are still approximately 89,900 deaths resulting from EC [16]. Thus, elucidating the pathogenesis, development, and tumorigenesis of EC is still important. Moreover, finding specific biomarkers for early diagnosis and seeking potential therapeutic targets are also urgent.

Accumulating evidence has inferred that centrosome amplification is closely linked to cell division disorder, chromosomal instability, and tumor occurrence [3–5, 12, 13, 17, 18]. However, no study has reported the relationship between EC progression and centrosome amplification. In addition, KIFC1, an essential centrosome clustering molecule, was reported to be overexpressed in various cancers [3–5], especially EC [6, 7]. Therefore, exploration of the linkage of KIFC1 and centrosome amplification is of great significance for the investigation of new therapeutic targets for tumors. A previous study showed that KIFC1 was overexpressed in lung cancer tissues, and the high KIFC1 mRNA expression subgroup had worse overall survival and progression-free survival [19]. Similarly, our data also showed that increased KIFC1 expression was negatively related to the overall survival of EC patients based on the database, and robust centrosome amplification was markedly observed in EC specimens.

As we all know, the centrosome is responsible for catalyzing microtubules, which are involved in the assembly of the mitotic spindle, and finally mediates the cell cycle progression [18]. Recently, centrosome amplification was recognized as a marker for cancer development [20], such as in Pca [4], GC [12], OC [3], and breast cancer [5]. We further investigated the effect of KIFC1 on centrosome amplification, cell cycle, and genomic stability. γ-tubulin and α-tubulin are the known markers for centrosome amplification. In the present study, KIFC1 overexpression markedly increased the accumulation of γ-tubulin and α-tubulin and the percent of centrosome amplification in EC cell lines, indicating that KIFC1 promoted abnormal centrosome amplification in EC. Moreover, our results showed that KIFC1 overexpression markedly induced chromosomal instability and cell cycle progression at G2/M indicated by increased mRNA and protein levels of Cyclin A2 and Cyclin B1. During the process of G2 and M phases, CDK1 and CDC2 act as the cyclin-dependent kinases that phosphorylate select proteins to drive cell cycle progression [21]. CDK1 and CDC2 can form complexes with Cyclin A2 (major in G2) and Cyclin B1 (major in M), which further phosphorylated corresponding substrates and promote cell cycle transition from the G2 phase to the M phase [22]. Corresponding to the increased levels of Cyclin A2 and Cyclin B1, the protein expressions of CDK1 and CDC2 were also upregulated in KIFC1-ovexpressed cells in our study. One previous study also reported that KIFC1 overexpression induced amplified and clustered centrosomes during interphase and mitosis in PCa cells, and increased the cell population in S and G2/M phases [4]. Other literature also indicated that higher expression of KIFC1 in OC increased the expression of genes related to centrosome amplification [3]. In addition, one study showed that KIFC1 knockdown can inhibit CDC2 expression and arrest cells in the G2/M phase [19], which was in accord with our results. Together, KIFC1 induced centrosome amplification and chromosomal instability, promoted cell cycle progression, and then contributed to EC progression.

Centrosomes replicate one per cell cycle during mitosis, regulated by serine/threonine protein kinase PLK4 [12, 13]. Previous studies have inferred that PLK4 inhibition caused centrosome depletion, leading to delayed acentrosomal spindle assembly [23, 24], while PLK4 stabilization resulted in centrosome amplification and cancer development [12, 24–26]. More importantly, PLK4 was enriched in various cancers and promoted tumor proliferation, invasion, migration, and metastasis [24, 25, 27–29]. A new study indicated that higher PLK4 protein expression was negatively associated with tumor characteristics and shortened survival in EC patients [30], suggesting the promoting effect of PLK4 on EC progression. In line with the above studies, PLK4 was also upregulated in EC cell lines in this study. Furthermore, PLK4 overexpression contributed to centrosome amplification, as indicated by the accumulation of γ-tubulin and α-tubulin, and EC progression, as indicated by increased cell survival, proliferation, migration, and invasion. Proteins including PLK4, Centrin2, and CEP215 are known markers for centrosome amplification [4, 13, 26, 31], and the regulatory effect of KIFC1 on centrosome amplification was widely recognized [3, 4, 32]. Our results further exhibited that KIFC1 overexpression markedly increased PLK4 expression, and the contributing role of PLK4 in centrosome amplification and EC progression was neutralized by KIFC1 silencing, which proved that KIFC1 promoted centrosome amplification dependent on PLK4. Another study also showed that KIFC1 overexpression interacted with Centrin2 to promote the clustering of amplified centrosomes in PCa cells [4]. Together, KIFC1 contributed to EC progression through centrosome amplification-related protein PLK4 in our study.

Besides, we found that KIFC1 overexpression increased the protein stability of PLK4. Several findings have inferred that various post-translational modifications, including phosphorylation, ubiquitination, and glycosylation, impacted the stability of a series of proteins to regulate biological processes [33]. Among these, ubiquitination is pivotal in the cell cycle, immune response, and tumor occurrence [34]. Moreover, PLK4 deubiquitylation was reportedly involved in centrosome amplification in GC [12]. Similarly, our results also showed that ubiquitin-proteasome pathway inhibitor MG132 could reverse the decreased protein level of PLK4 by KIFC1 knockdown, while autophagy-lysosome pathway inhibitor CQ should have no impact. Moreover, the ubiquitination assay also showed that KIFC1 overexpression significantly inhibited PLK4 ubiquitination, which indicated that KIFC1 enhanced the protein stability of PLK4 by reducing its ubiquitination. However, whether other modifications participated in maintaining PLK4 stabilization was unknown and needed further investigation.

Ubiquitination is catalyzed by E1 activating enzymes, E2 conjugating enzymes, and E3 ligases [35]. Among these, E3 ligases transfer Ub from E2 ubiquitin-conjugating enzyme to specific substrate proteins, resulting in distinct outcomes, such as cell death, cell cycle progression, protein stability, and tumor recurrence [34–36]. TRIM family, with E3 ubiquitin ligase activities in the majority of its members, was reported to regulate multiple cellular processes and signaling pathways, especially human tumorigenesis [14, 37–39]. Up to now, about 80 TRIM proteins have been identified, and each TRIM was thought to target specific interactors and substrates [37]. Several members of the TRIM family were involved in the regulation of centrosome amplification and cancer progression. For example, TRIM8 interacted with KIFC1 to control bipolar spindle formation and chromosomal stability [40]. TRIM37 was considered a critical determinant of mitotic vulnerability to PLK4 inhibition [13]. On the one hand, TRIM37 inactivation, which contributed to PLK4 self-assembly into centrosome-independent condensates, improved acentrosomal mitosis [13]. On the other hand, increased TRIM37 enhanced the degradation of centrosomal component CEP192 to inhibit acentrosomal spindle assembly [13]. In addition, TRIM69-ablation in cancer cells leads to centrosome scattering and chromosome segregation defects [41]. In our study, KIFC1 overexpression was significantly inhibited, while PLK4 overexpression showed no impact on TRIM37 expression. In addition, Co-IP assays showed direct binding of PLK4 with TRIM37, not with KIFC1, which together indicated that PLK4 was the potential substrate of TRIM37. Moreover, TRIM37 expression was markedly increased in PLK4-overexpressed and KIFC1-knockdown EC cells compared with that in PLK4-overexpressed cells, and TRIM37 overexpression significantly increased PLK4 ubiquitination. Additionally, KIFC1 showed a positive correlation with PLK4 expression, while TRIM37 showed a negative correlation with KIFC1 and PLK4, which together verified that KIFC1 reduced TRIM37 expression to inhibit PLK4 ubiquitination and enhanced PLK4 stability that further induced centrosome amplification and tumor progression. Another study inferred that USP54, a novel deubiquitinating enzyme, also targeted PLK4 to attenuate centrosomal clustering and GC progression [12]. Controversial studies have reported that TRIM37 knockdown reduced the proliferation, clonogenicity, migration, and invasion ability of tumor cells and suppressed tumor growth in vivo [42–44], indicating the promoting effect of TRIM37 in PCa, OC, and gallbladder cancer progression. However, our data suggested that TRIM37 inhibited EC progression based on cell experiments and mouse models, which was reversed by KIFC1 overexpression. In this study, we first demonstrated that TRIM37 inhibited EC tumorigenesis in vitro and in vivo. The possible reason might be different cancer types and the involvement of specific substrates of TRIM37. Furthermore, the promoting effect of KIFC1 on EC progression was verified in our study, as previously reported [6, 7].

In conclusion, our finding demonstrated that KIFC1 reduced the expression of TRIM37, which inhibited ubiquitination degradation of PLK4 and maintained its stabilization, to further promote centrosome amplification and EC progression. Therefore, our study provided effective biomarkers and therapeutic targets for EC patients in the future.

## Method and materials

### Bioinformatic analysis

The RNA sequencing data of 548 EC samples (normal = 35, primary tumor = 546) and corresponding information in the Cancer Genome Atlas (TCGA) database were downloaded from the OncoLnc website (http://www.oncolnc.org/), and the gene expression of KIFC1 was further analyzed in our study. The correlation analysis between KIFC1 expression and survival in EC samples was conducted in the Kaplan-Meier-plotter dataset (http://kmplot.com/analysis/).

### Tissue samples

Five human EC specimens, along with matched adjacent normal tissues, were collected from The Quzhou Affiliated Hospital of Wenzhou Medical University, Quzhou People’s Hospital. All the procedures were by the principles of the Ethics Committee of The Quzhou Affiliated Hospital of Wenzhou Medical University, Quzhou People’s Hospital (Approval no. 2022-113). Before the sample collection, all patients were informed, and the consent was signed for analysis and publication.

### H & E, immunohistochemistry (IHC) and immunofluorescence (IF) staining

The clinical EC specimens and mice tissues were fixed with 4% paraformaldehyde (PFA) for 24 h at room temperature, and sent to Servicebio (Wuhan, China) for H & E staining, IHC, and IF analysis according to their instructions. The antibodies anti-KIFC1 (1:200, No. 20790-1-AP, Proteintech), anti-PLK4 (1:200, No. 12952-1-AP, Proteintech), anti-TRIM37 (1:200, No. 13037-1-AP, Proteintech) and anti-Ki-67 (1:200, 27309-1-AP, Proteintech) were used for IHC staining. The antibodies anti-KIFC1 (1:50, No. 20790-1-AP, Proteintech), anti-γ-tubulin (1:200, No. 66320-1-Ig, Proteintech), and anti-α-tubulin (1:200, No. CL594-66031, Proteintech) were used for IF staining. DAPI (CAS#28718-90-3, Sigma) was used for nucleus staining. Images of stained specimens were captured using a Zeiss UV LSM 510 microscope.

### Cell lines and reagents

The following human EC cell lines, including HEC-1A, HEC-1A-Luc, and Ishikawa cells, normal human endometrial endothelial cell line (hEEC), and HEK293T were purchased from American Type Culture Collection (ATCC). The Ishikawa cells were maintained in modified Eagle’s medium (MEM, Gibco) supplemented with 5% fetal bovine serum (FBS, Sigma-Aldrich) and 2% penicillin/streptomycin (100 U/mL, Biological Industries). The HEC-1A, HEC-1A-Luc, and HEK293T cells were cultured in Dulbecco’s Modified Eagle Medium (DMEM, Gibco) basic supplemented with 10% FBS and 1% penicillin/streptomycin. In addition, hEEC cells were inoculated in Roswell Park Memorial Institute (RPMI)-1640 (Invitrogen) with 10% FBS and 1% penicillin/streptomycin. All cell lines were tested twice a month for mycoplasma by the LookOut Mycoplasma PCR Detection Kit (Sigma-Aldrich) and incubated at 37 °C in a humidified atmosphere of 5% CO_2_. Cycloheximide (CHX, Cat#S7418), proteasome inhibitor MG132 (Cat#S2619), and chloroquine diphosphate (CQ, Cat#S4157) were purchased from Selleck Chemicals (Houston, TX, USA).

### Plasmid construction, lentiviral short-hairpin RNAs, and cell transfection

According to our previous study, full-length cDNA encoding human KIFC1, PLK4, and TRIM37 were PCR-amplified and cloned into pcDNA3.1 vector (GenScript) to upregulate their expressions and then were transfected into HEC-1A and Ishikawa cells for exosmotic study. PcDNA 3.1 vector (Vector), pcDNA3.1-KIFC1 (OE-KIFC1), pcDNA3.1-PLK4 (OE-PLK4), and pcDNA3.1-TRIM37 (OE-TRIM37) were originated from GenScript (Shanghai, China) according to their instructions [45]. ShRNA oligonucleotide sequences targeting human KIFC1 were synthesized by GenScript, and the sequence was: shKIFC1, 5’-GGACTTAAAGGGTCAGTTATG-3’. Nonsense shRNA was used as a negative control (shNC: 5’-TTCTCCGAACGTGTCACGTAA-3’).

EC cells were cultured to 50% confluence in 6 cm dishes and transfected with different vectors using Lipofectamine 3000 (Invitrogen) to overexpress or knock down the expression of related genes. As in the previous study, the cells were harvested after 24–48 h for transfection and used for later analysis [45].

For the animal study, the overexpressing vectors were transfected into HEK293T cells with lentiviruses. Finally, the whole lentiviruses were collected and delivered into HEC-1A and HEC-1A-Luc cells, which were selected with 3 μg/mL puromycin for one month to establish stable overexpressing cells. Normal cells or stable overexpressing cells were cultured and used for nude mice.

### Western blot analysis

RIPA lysis buffer (Beyotime Biotechnology, Shanghai) supplemented with a protease inhibitors cocktail (Roche, Switzerland) was used to collect total proteins from human EC cells (HEC-1A, HEC-1A-Luc, and Ishikawa cells) and normal hEEC. Then, the protein concentrations were determined by a standard BCA protein assay kit (KeyGEN BioTECH). Equal proteins (approximately 30 μg) were subjected to SDS-PAGE and then transferred to polyvinylidene fluoride (PVDF) membranes (Bio-Rad Laboratories). The blots were then blocked in 5% nonfat dry milk solution (Beyotime Biotechnology, Shanghai) at room temperature for 1 h. Subsequently, the blots were incubated with the following primary antibodies: KIFC1 (1:1000, No. 20790-1-AP, Proteintech), γ-tubulin (1:5000, No. 66320-1-Ig, Proteintech), p-H3 (S10) (1:1000, #9701, Cell Signaling Technology), Cyclin A2 (1:1000, #67955, Cell Signaling Technology), Cyclin B1 (1:1000, #4138, Cell Signaling Technology), CDK1 (1:10000, No. 10122-1-AP, Proteintech), CDC2 (1:1000, #77055, Cell Signaling Technology), PLK4 (1:1000, No. 12952-1-AP, Proteintech), TRIM37 (1:15.00, No. 13037-1-AP, Proteintech), Ub (1:1000, ab134953, Abcam), Myc (1:1000, ab32, Abcam), HA (1:1000, ab1424, Abcam), His (1:1000, ab18184, Abcam) and GAPDH (1:10000, ET1601-4, Huabio) at 4 °C overnight. After washing 3 times and inoculating with secondary antibodies for 1 h at room temperature, protein bands were finally visualized using an ECL luminescence reagent (Tanon, China). The bands were then quantified by ImageJ software (NIH, Bethesda).

### RNA isolation and quantitative reverse transcription polymerase chain reaction (qPCR)

Total RNA from cultured cells was isolated using TRIzol reagent (Invitrogen) according to the manufacturer’s protocol. Then, the purity and concentration of RNA were determined by NanoDrop 200 C (Thermo Fisher Scientific, USA). Subsequently, 2 μg of RNA was reverse transcribed into cDNA using RevertAid RT Reverse Transcription Kit (Thermo Fisher Scientific, USA). qPCR was conducted with FastStart Universal SYBR Green master mix (Roche, Mannheim, Germany) via StepOnePlus Real-Time PCR system (Applied Biosystems, Foster City, USA) according to the manufacturer’s protocols. Each sample was run in triplicate and normalized to the reference GAPDH gene expression. The relative mRNA expression of target genes was calculated using the 2^−△△Ct^ method. The gene-specific primers were designed via NCBI Primers BLAST and listed as follows:

KIFC1-F: 5′-ACCCAGTTCTCTTCCACTGC-3′;

KIFC1-R: 5′-GGGATGGAACTCTTGGGTGG-3′.

PLK4-F: 5′-CGGAAGGTGTCAGGGAGAAC-3′;

PLK4-R: 5′-GATCTTCTCCCCGATGCAGG-3′.

TRIM37-F: 5′-GGAGAAATTGCGGGATGCAC-3′;

TRIM37-R: 5′-CTGTCAGCCAGCGCCTAATA-3′.

Cyclin A2-F: 5′-GCACTGGTGGTCTGTGTTCT-3′;

Cyclin A2-R: 5′-TGGATGCCAGTCTTACTCATAGC-3′.

Cyclin B1-F: 5′-ATGTGCCCCTGCAGAAGAAG-3′;

Cyclin B1-R: 5′-TGGTCTCCTGCAACAACCTG-3′.

GAPDH-F: 5′-CTGGGCTACACTGAGCACC-3′;

GAPDH-R: 5′-AAGTGGTCGTTGAGGGCAATG-3′.

### Cell counting kit (CCK)-8 assay

Cell viability was measured by the CCK-8 reagent (Dojondo Laboratories, Kumamoto, Japan) according to the manufacturer’s instructions. Briefly, HEC-1A and Ishikawa cells were seeded onto 96-well plates at 2 × 10^3^ cells per well overnight and then transfected with various plasmids for 24 h. Subsequently, each well was filled with 10 μL of CCK-8 solution and incubated for an additional 4 h. The absorbance of each well was determined at 450 nm using synery^TM^H1 Muti-Mode Reader spectrophotometry (Thermo Fisher Scientific).

### Colony formation assay

The colony formation assay was performed as previously described [12]. Briefly, HEC-1A and Ishikawa cells were counted and seeded into a six-well plate (1000 cells/well). The cells were cultured in the corresponding media for two weeks. Finally, the cells were washed with PBS, fixed with methanol, and stained with 0.1% crystal violet in 20% methanol for 20 min, and the number of colonies in each well was photographed and counted.

### Transwell invasion and migration assays

The cells (HEC-1A and Ishikawa) were transfected with different plasmids for 24 h. Then, treated cells (1 × 10^5^/well) were suspended in a serum-free medium and then seeded in the upper layer of a trans-well chamber (six-well), which was precoated with (invasion assay) or without (migration assay) 50 μL Matrigel (Becton Dickinson). A complete growth medium (700 μL) with 10% FBS was added to the lower chamber. The cells were allowed to invade for another 24 h. After 48 h of incubation, the invaded cells on the lower surface of the membrane were then fixed with 4% paraformaldehyde (PFA) for 30 min and stained with 0.1% crystal violet solution for 15 min. Finally, six fields were randomly selected to calculate the cell area under a light microscope (Olympus Corporation, Tokyo, Japan).

### TUNEL staining

According to the previous study [46], cell apoptosis in mouse tumor tissues was measured by TUNEL staining. Briefly, tumor tissues were collected and fixed in a 4% PFA solution immediately after excising and embedded in paraffin. Then, the samples were sliced, and apoptotic cells were detected using the TUNEL assay kit according to the manufacturer’s instructions. DAPI was used for nucleus staining. For each slide, six fields were randomly chosen and pictured.

### Co-IP

For the Co-IP assay, the Myc-KIFC1 and His-TRIM37 plasmids, and HA-Ub were synthesized by GenScript according to their instructions. HEK293T cells were transfected with different plasmids for 48 h. Then, cells were centrifuged, harvested, and resuspended in 2 × volume of IP lysis buffer (Pierce) supplemented with protease inhibitors cocktail (Roche, Switzerland) on ice for 10 min. Thereafter, the samples were subjected to immunoprecipitation with anti-PLK4, anti-HA, normal IgG antibody, and protein G-Agarose beads (Roche Diagnostics Ltd, Shanghai, China) overnight at 4 °C. The beads were washed, resuspended in an SDS sample buffer, and analyzed by western blot analysis.

To verify whether KIFC1, PLK4, and TRIM37 interact with each other, HEC-1A and Ishikawa cells were harvested and resuspended in 2 × volume of IP lysis buffer supplemented with protease inhibitors cocktail on ice for 10 min. Thereafter, the samples were subjected to immunoprecipitation with anti-PLK4, anti-KIFC1, normal IgG antibody, and protein G-Agarose beads overnight at 4 °C. The beads were washed, resuspended in an SDS sample buffer, and analyzed by western blot analysis (KIFC1, TRIM37, PLK4, and GAPDH). The input was set as samples without IP, which was directly analyzed by western blot analysis.

### Ubiquitination assay

HEK293T cells were transiently transfected with specific plasmids and HA-Ub for 48 h. Cell lysates were collected, and the ubiquitination assay was performed with a Ubiquitylation Assay Kit (Abcam) according to the manufacturer’s protocol.

### In vivo xenograft experiments

The effect of KIFC1 and TRIM37 on the EC progression was accessed using an in vivo nude mice model. The mouse experiments were approved by the Ethics Committee of The Quzhou Affiliated Hospital of Wenzhou Medical University, Quzhou People’s Hospital (Approval no. xmsp2022-0913). 36 female BALB/c nude mice (6 weeks old) were purchased from the Laboratory Animal Center of the Chinese Academy of Sciences (Shanghai, China) for study. Mice were maintained under specific pathogen-free (SPF) conditions under a 12-h light/dark cycle for 7 continuous days before use. The mice were randomly divided into three groups: Vector, OE-TRIM37, and OE-TRIM37 + OE-KIFC1, with 12 mice per group. A total of 1 × 10^7^ HEC-1A cells that stably overexpressed vector, TRIM37, or TRIM37 and KIFC1 were collected and resuspended in 100 μL PBS. Then, the cells were subcutaneously injected into the right flank of mice, individually. The experiment lasted for 4 weeks. Tumor length and width were recorded every week after inoculation, and the tumor volume was calculated. The isolated tumors were photographed, weighed, and subjected to further pathological analysis. IHC staining of tumors was used to access the contents of TRIM37, KIFC1, and PLK4, while the proliferation was quantified by calculating the Ki-67-positive cells. For the lung metastasis model, HEC-1A-Luc cells (1 × 10^6^) stably overexpressing TRIM37 or TRIM37 and KIFC1 were injected into the tail vein of female BALB/c nude mice. Then, mice were anesthetized with 1% pentobarbital sodium and intraperitoneally injected with luciferin (Promega, P1043, 100 μg/kg of body weight). After 15 min, luciferase activity was evaluated using the IVIS Spectrum In vivo Imaging System (Perkin Elmer, Waltham, MA). Mice were euthanized 28 days after cell injection, and their lungs were extracted. The amount of lung metastasis was quantitated using the IVIS imaging system and confirmed by H & E staining. The investigator was blinded to the race of the cell lines throughout the whole process (from the injection of cell lines to the completion of all the analyses).

### Statistical analysis

All data were presented at the mean ± SEM. To analyze the differences between two or more groups, a two-tailed Student’s *t*-test and ANOVA were used. The overall survival (OS) curves were plotted using the Kaplan–Meier method and compared using the log-rank test. The statistical significance of differences among groups was indicated by asterisks (*/^*P* < 0.05, **/^^*P* < 0.01, and ***/^^^*P* < 0.001). Statistical analysis was performed using GraphPad Prism 9.0 (GraphPad Software, La Jolla, CA). For biological experiments, data were obtained from at least 3 independent experiments. The Pearson correlation analysis in EC patients between the mRNA expressions of PLK4, KIFC1, and TRIM37 was conducted.

## Supplementary information

supplementary legends

SUPPLEMENTAL MATERIAL

original wb

## Data Availability

The authors declare that all data supporting the findings of this study are available within the paper, and any raw data can be obtained from the corresponding author upon request.
