# Supplementary material for: KIFC1 depends on TRIM37-mediated ubiquitination of PLK4 to promote centrosome amplification in endometrial cancer
Source: Cell Death Discov. 2024 Sep 30;10:419. doi: 10.1038/s41420-024-02190-1 (PMC11442630; doi:10.1038/s41420-024-02190-1)
Supplement: Supplementary file 1 — supplementary legends [file 41420_2024_2190_MOESM1_ESM.docx]

**Figure S1.** (A) The relative fluorescence intensities of KIFC1 and γ-tubulin in EC specimens (1-5) and adjacent normal tissues (6-10) of figure 1E; (B) The percent of centrosome amplification of figure 2A; (C) The mRNA expressions of Cyclin A2 and Cyclin B1 in KIFC1-overexpressed EC cells; (D) The protein expressions of CDK1 and CDC2 in KIFC1-overexpressed EC cells; (E) The percent of centrosome amplification of figure 3G; (F) Interaction analysis of KIFC1 and TRIM37 with PLK4 following PLK4 immunoprecipitation; (G) Interaction analysis of KIFC1 with TRIM37 following TRIM37 immunoprecipitation; (H) The Pearson correlation analysis in EC patients between the mRNA expression of PLK4, KIFC1 and TRIM37. **P* < 0.05, ***P* < 0.01, #*P* < 0.05, ##*P* < 0.01.
