# Supplementary figures and images for: KIFC1 depends on TRIM37-mediated ubiquitination of PLK4 to promote centrosome amplification in endometrial cancer
Source: Cell Death Discov. 2024 Sep 30;10:419. doi: 10.1038/s41420-024-02190-1 (PMC11442630; doi:10.1038/s41420-024-02190-1)

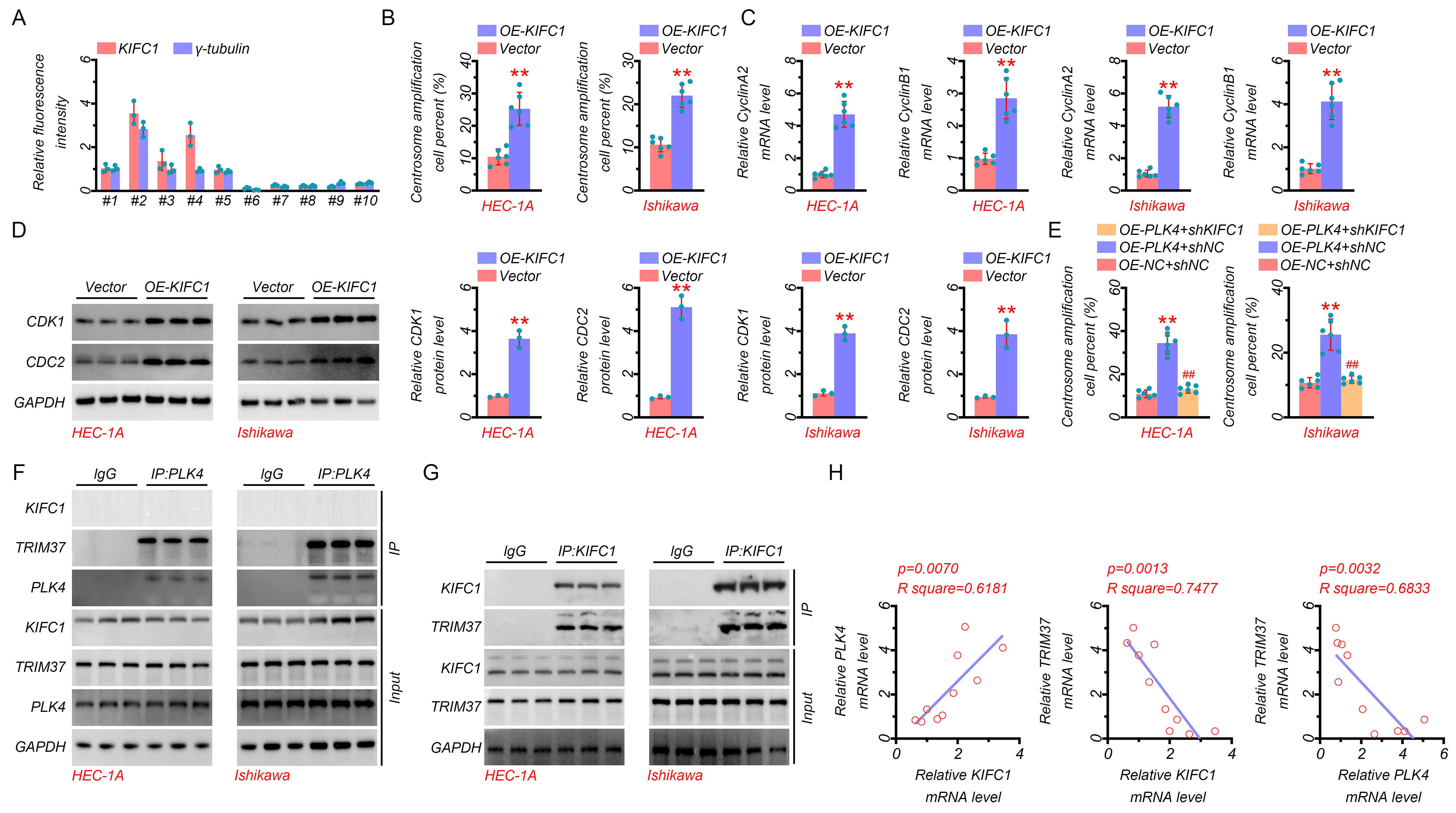

Supplement: Supplementary file 2 — SUPPLEMENTAL MATERIAL [file 41420_2024_2190_MOESM2_ESM.jpg]
