# Supplementary material for: KIFC1 depends on TRIM37-mediated ubiquitination of PLK4 to promote centrosome amplification in endometrial cancer
Source: Cell Death Discov. 2024 Sep 30;10:419. doi: 10.1038/s41420-024-02190-1 (PMC11442630; doi:10.1038/s41420-024-02190-1)

Figure 1 C

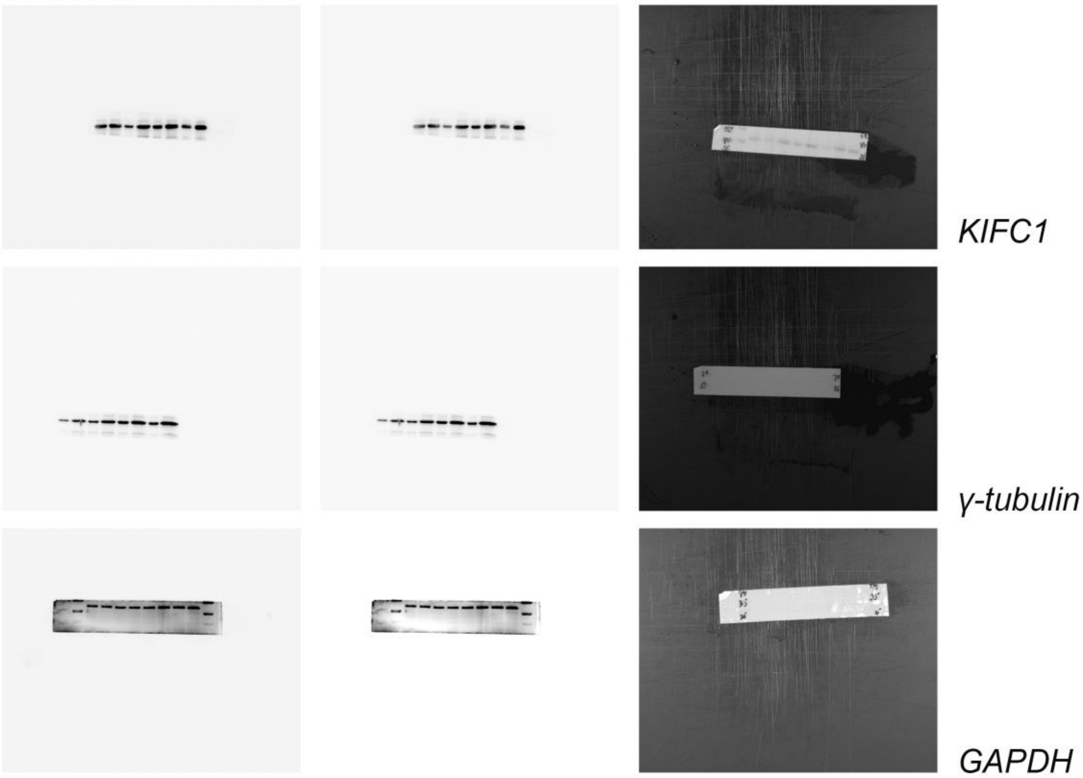

Figure 2 B-HEC-1A

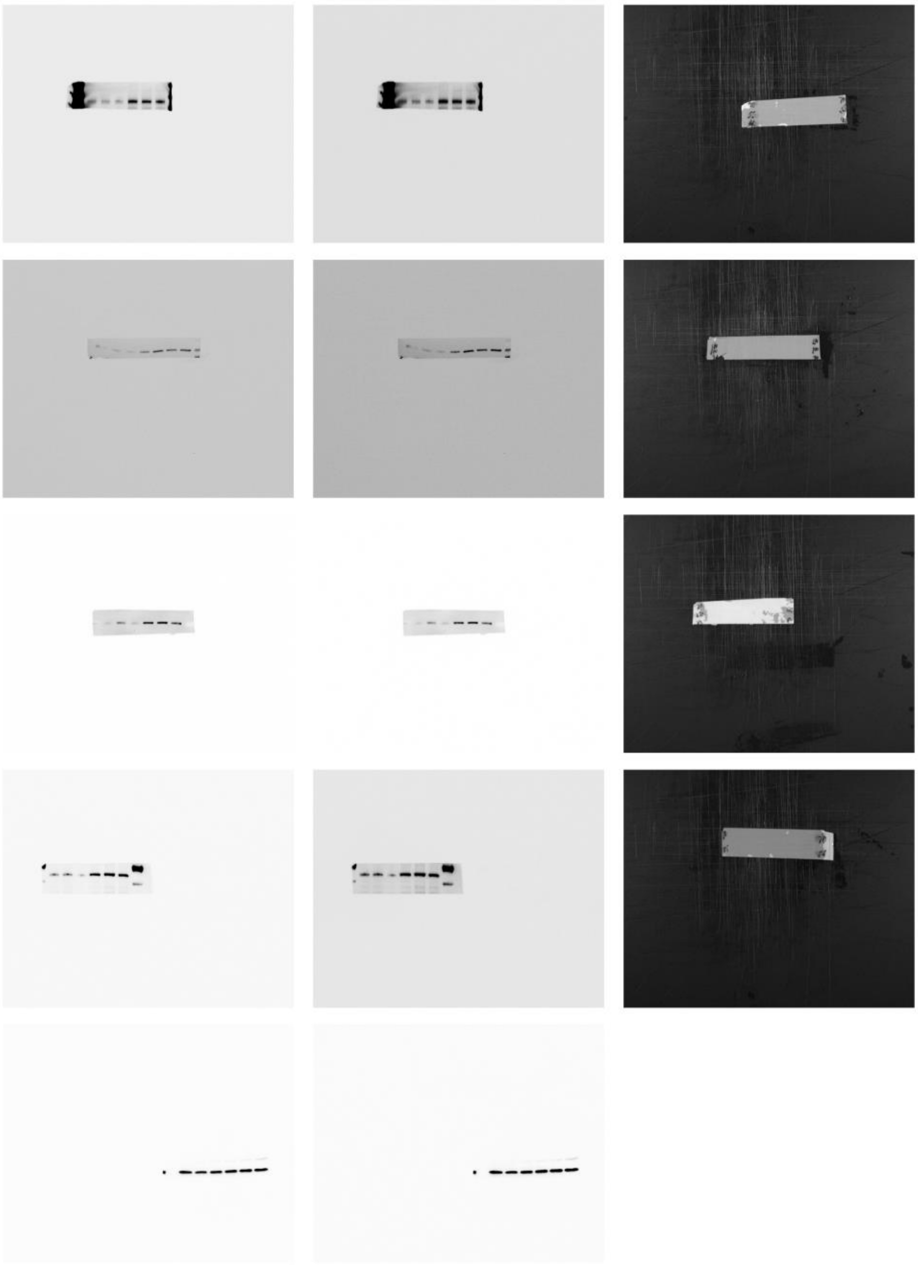

KIFC1

p-H3

Cyclin A2

Cyclin B1

GAPDH

Figure 2 B-Ishikawa

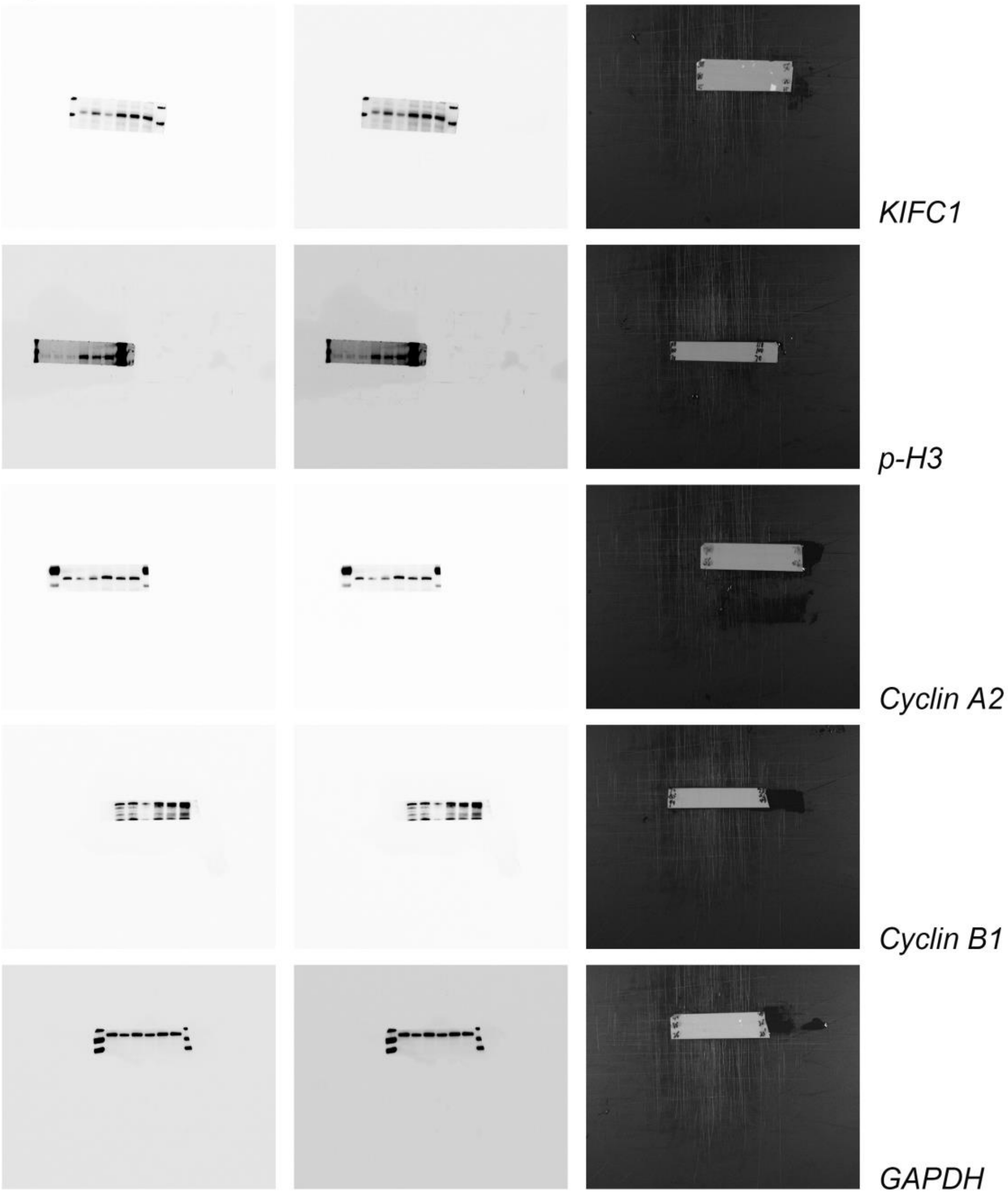

Figure 3 B

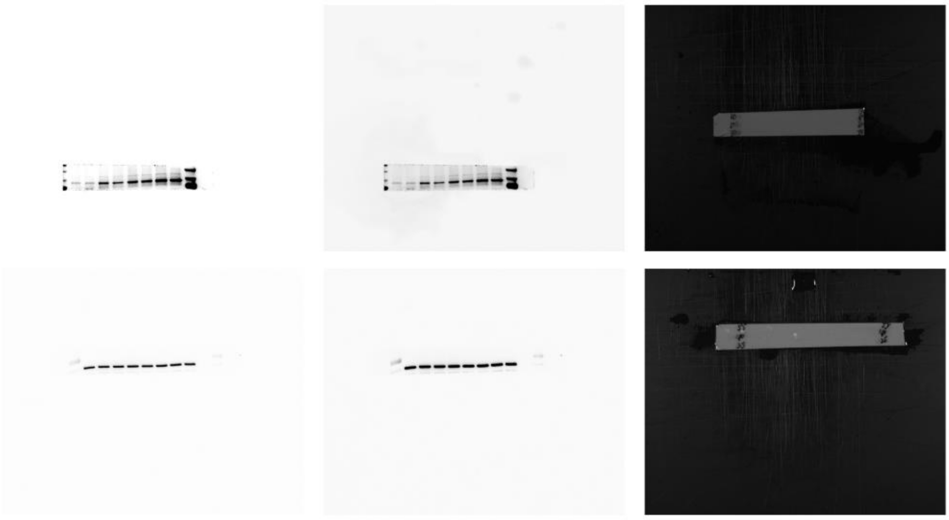

PLK4

GAPDH

Figure 3 D-HEC-1A

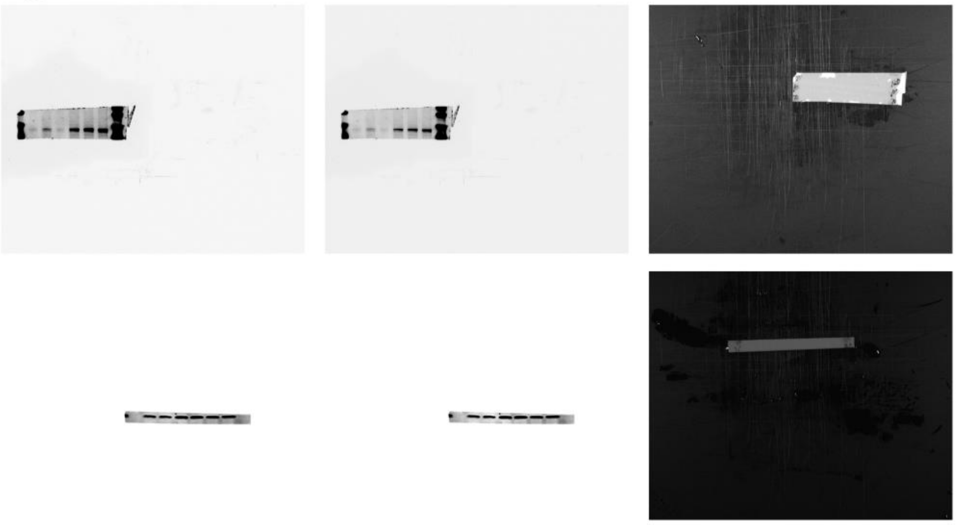

PLK4

GAPDH

Figure 3 D-Ishikawa

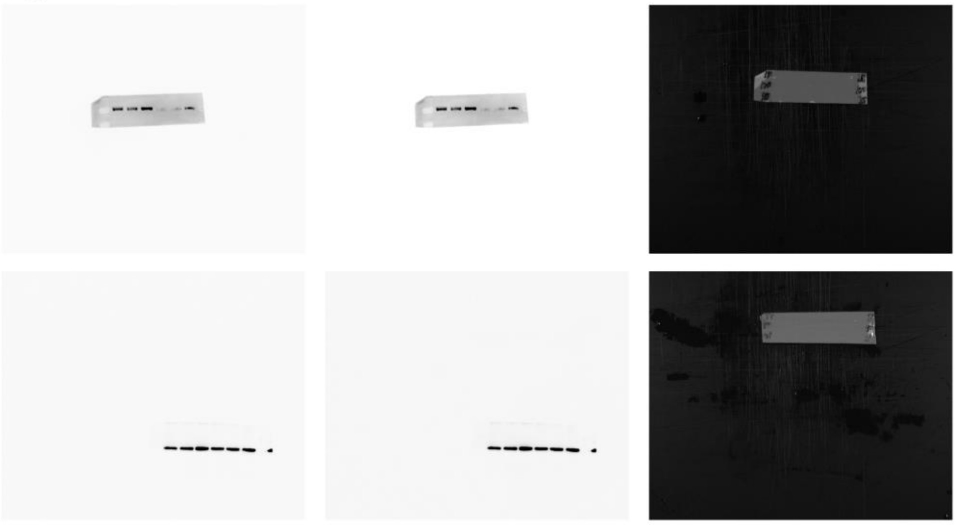

PLK4

GAPDH

Figure 3 F-HEC-1A

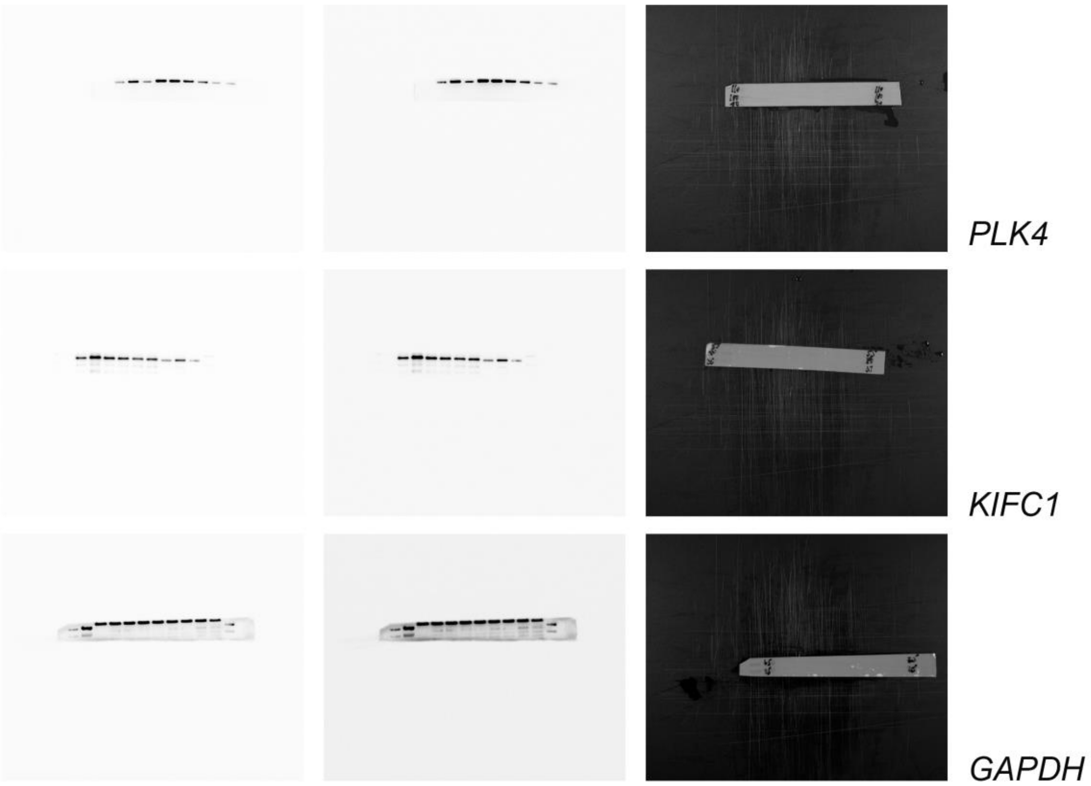

Figure 3 F-Ishikawa

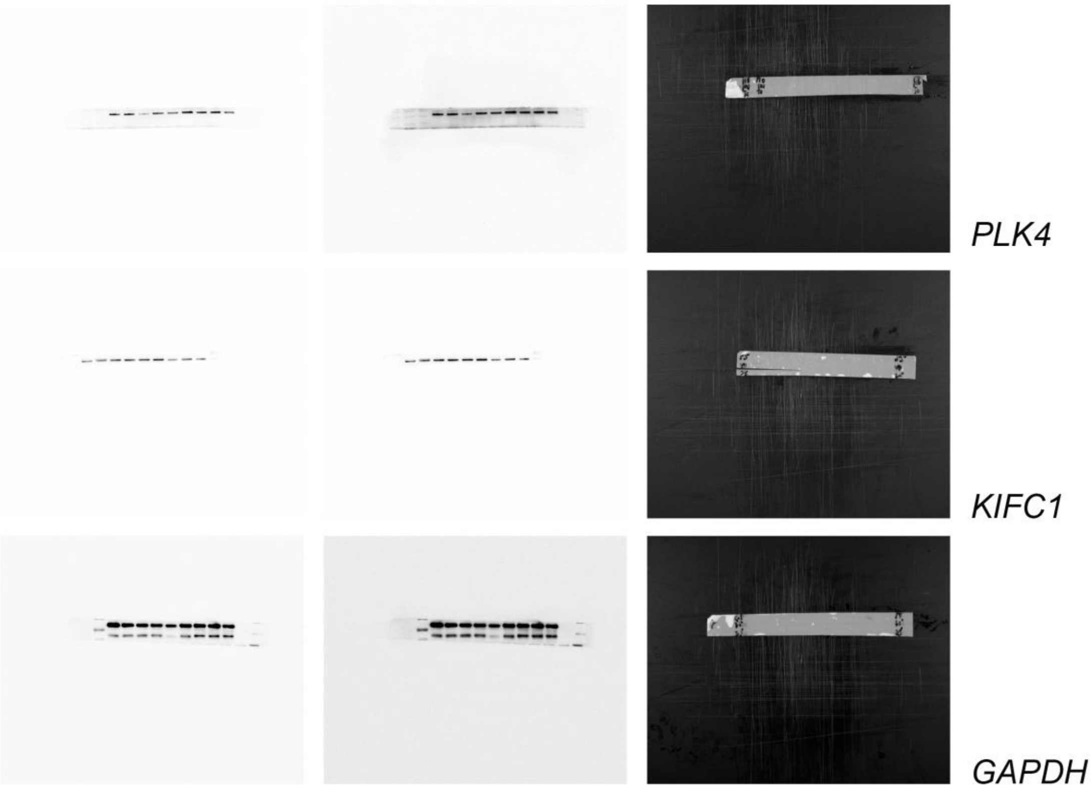

Figure 5 A-HEC-1A

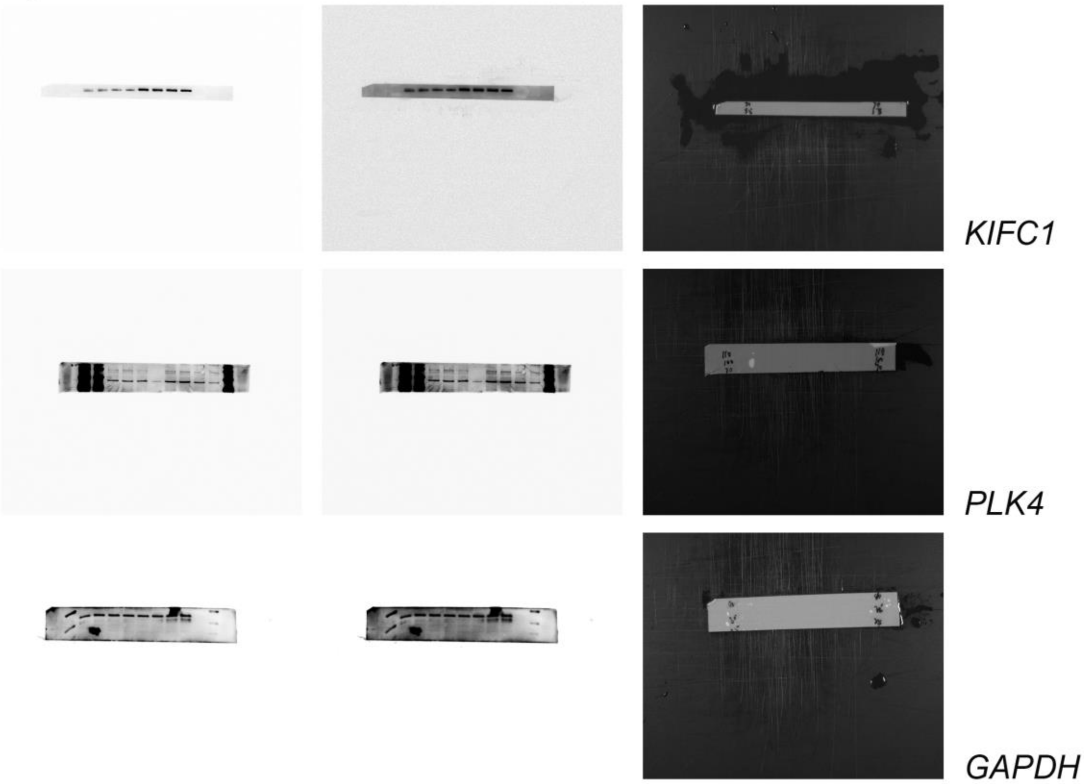

Figure 5 A-Ishikawa

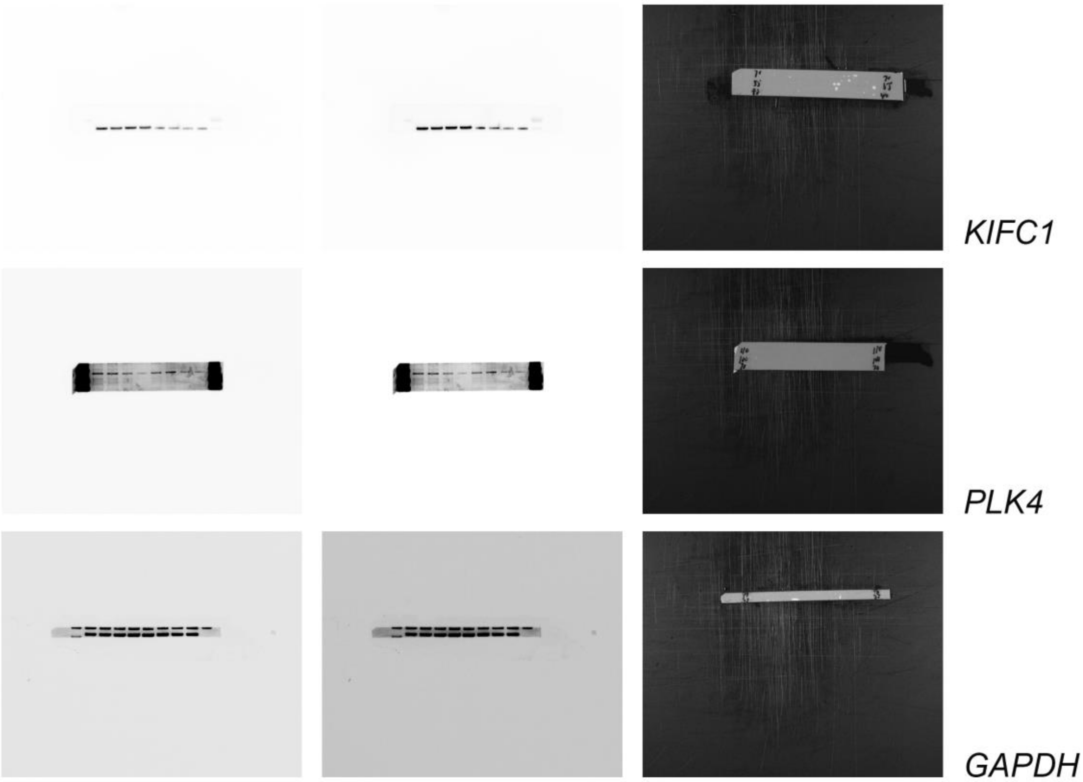

Figure 5 B-HEC-1A

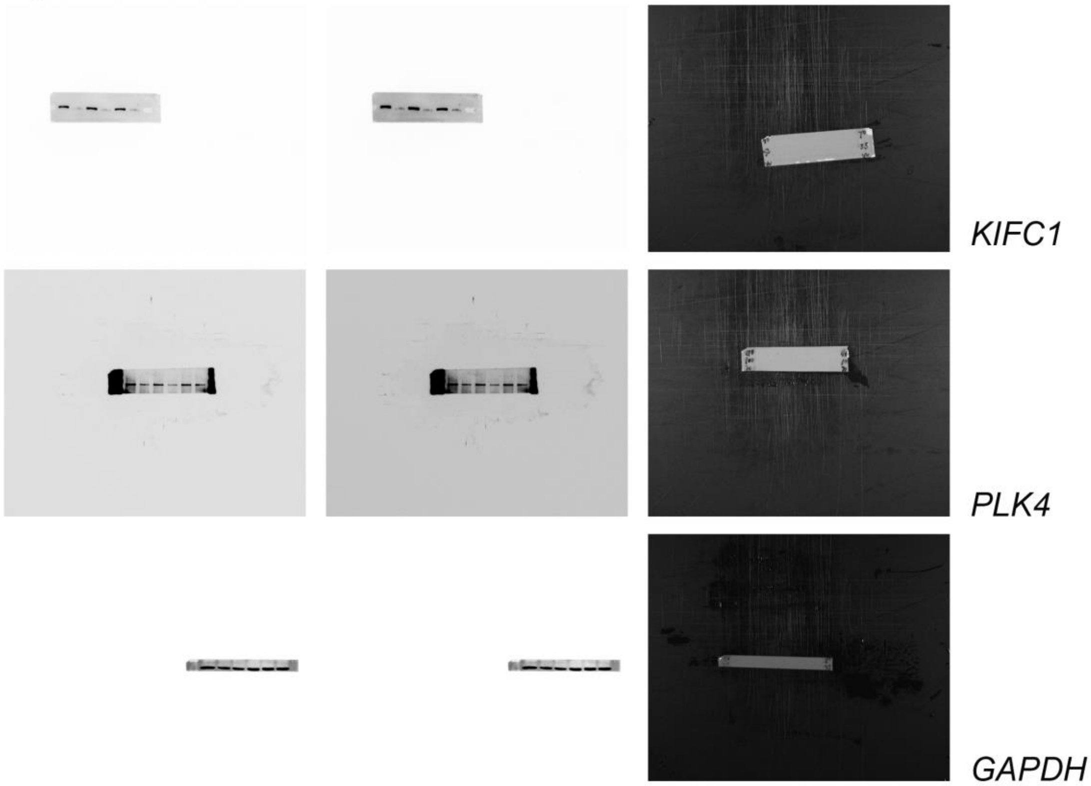

Figure 5 B-Ishikawa

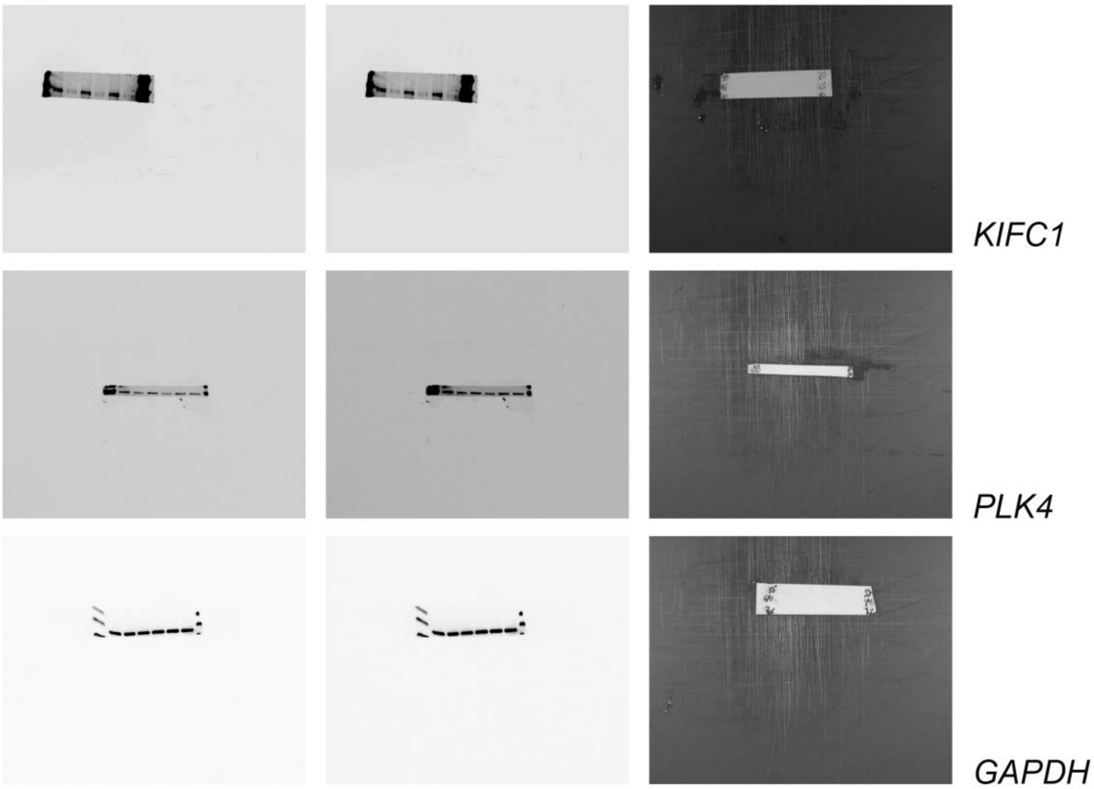

Figure 5 C

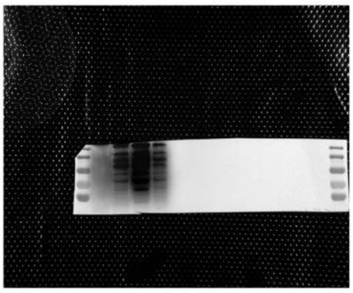

Ub

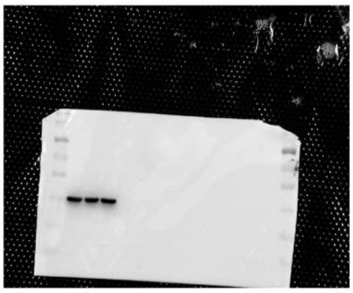

PLK4

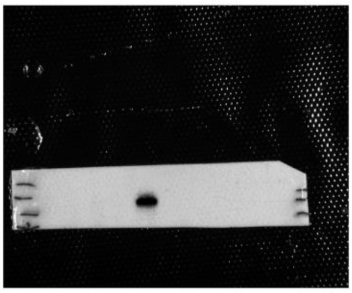

KIFC1

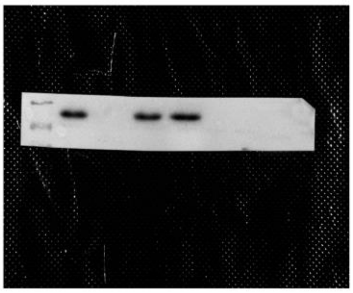

PLK4

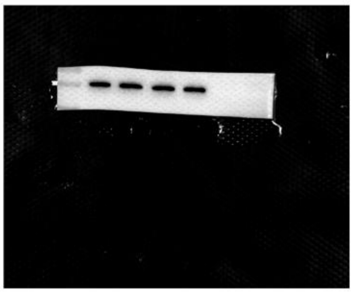

GAPDH

Figure 6 A-HEC-1A

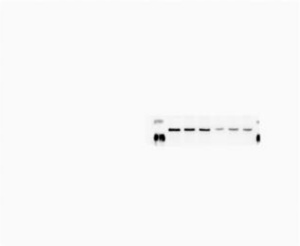

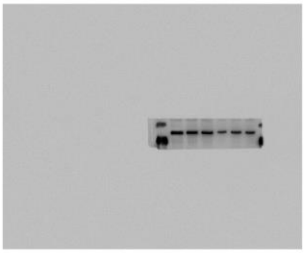

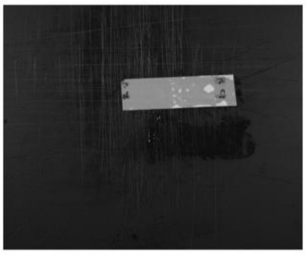

TRIM37

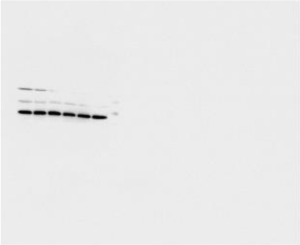

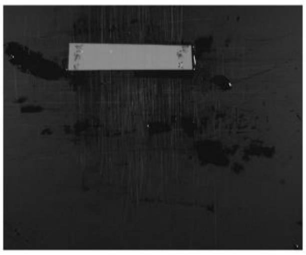

GAPDH

Figure 6 A-Ishikawa

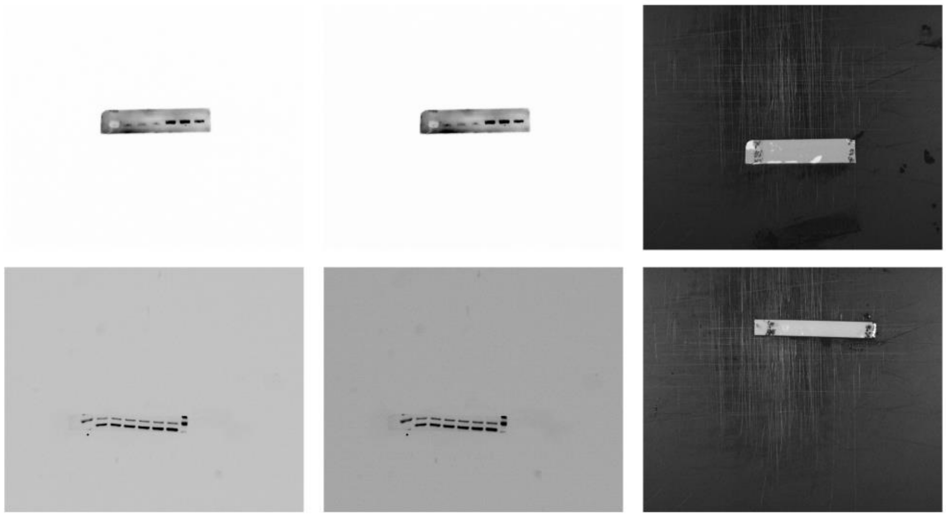

TRIM37

GAPDH

Figure 6 B-HEC-1A

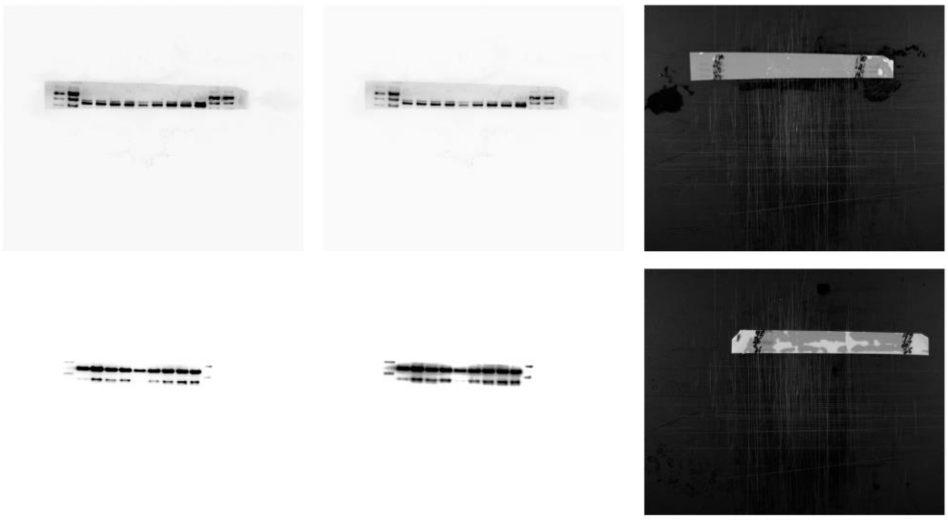

TRIM37

GAPDH

Figure 6 B-Ishikawa

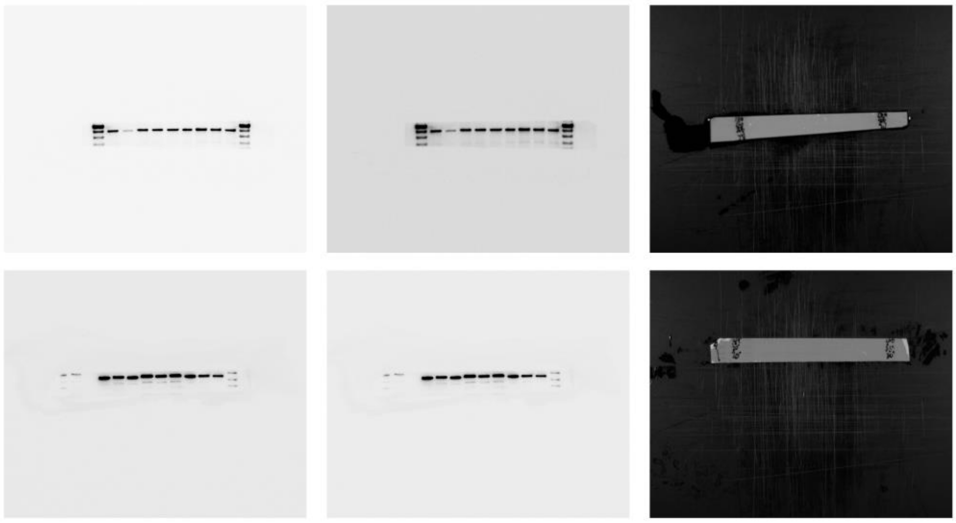

TRIM37

GAPDH

Figure 6 C

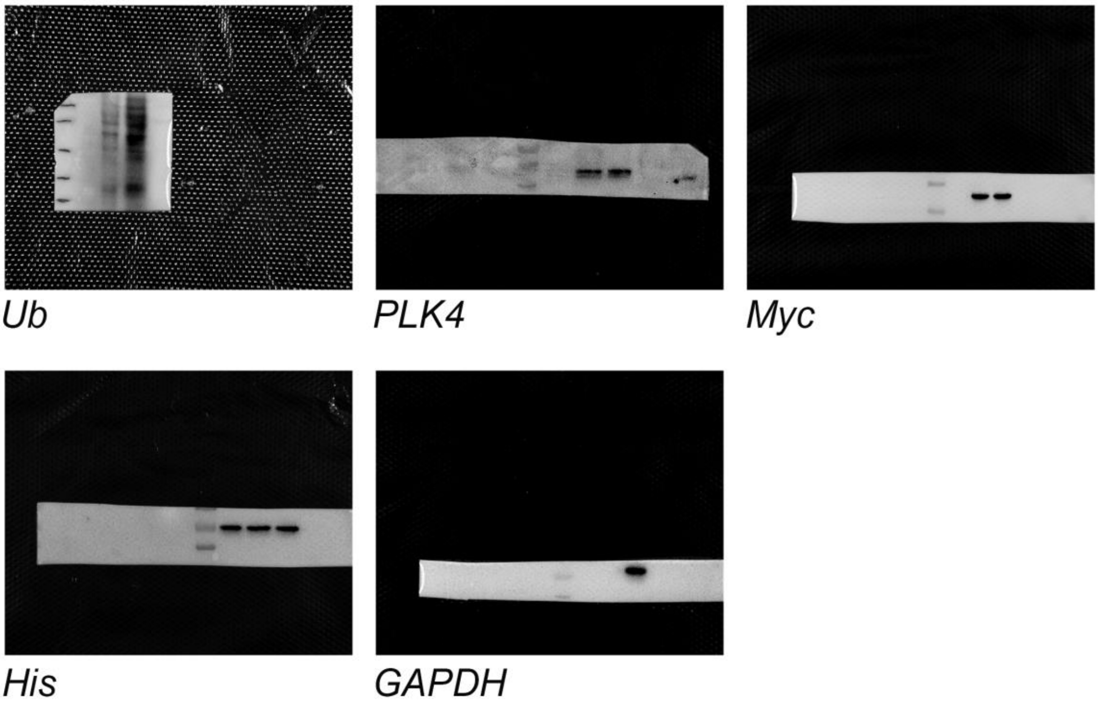

Figure 6 H-HEC-1A

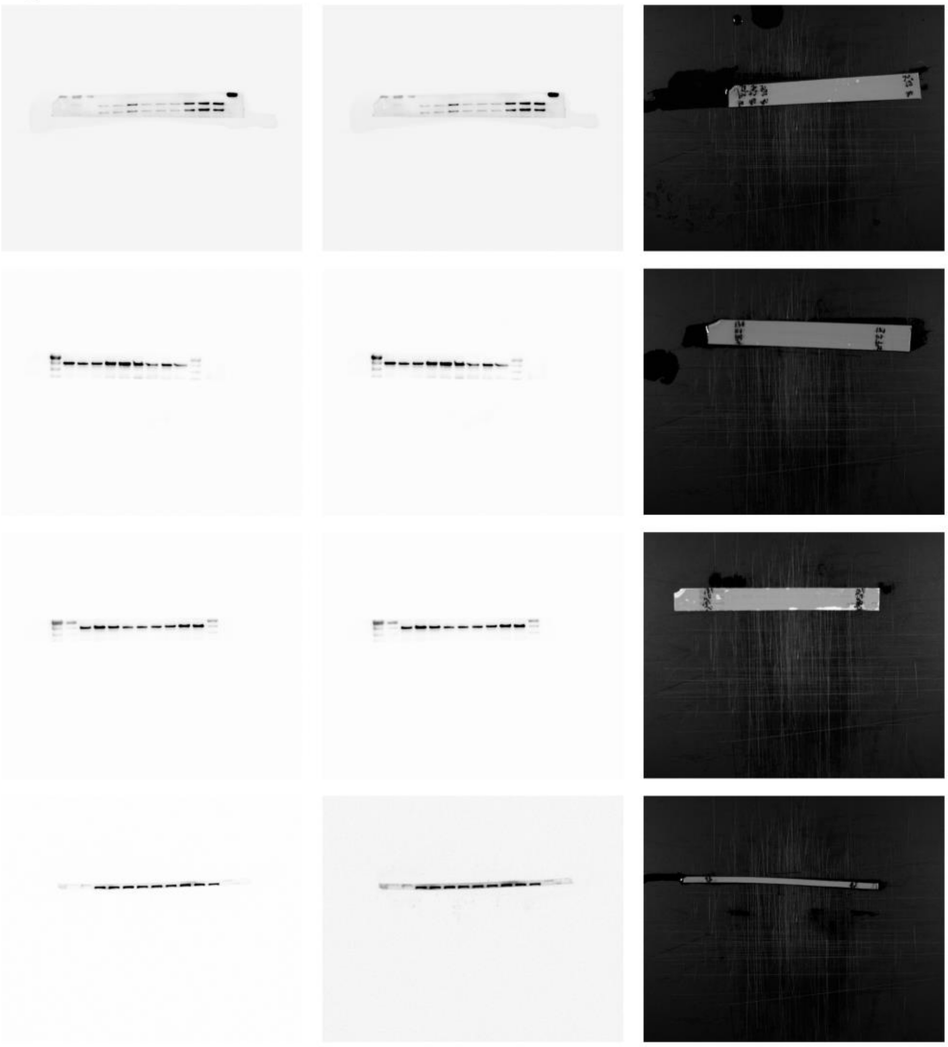

KIFC1

TRIM37

PLK4

GAPDH

Figure 6 H-Ishikawa

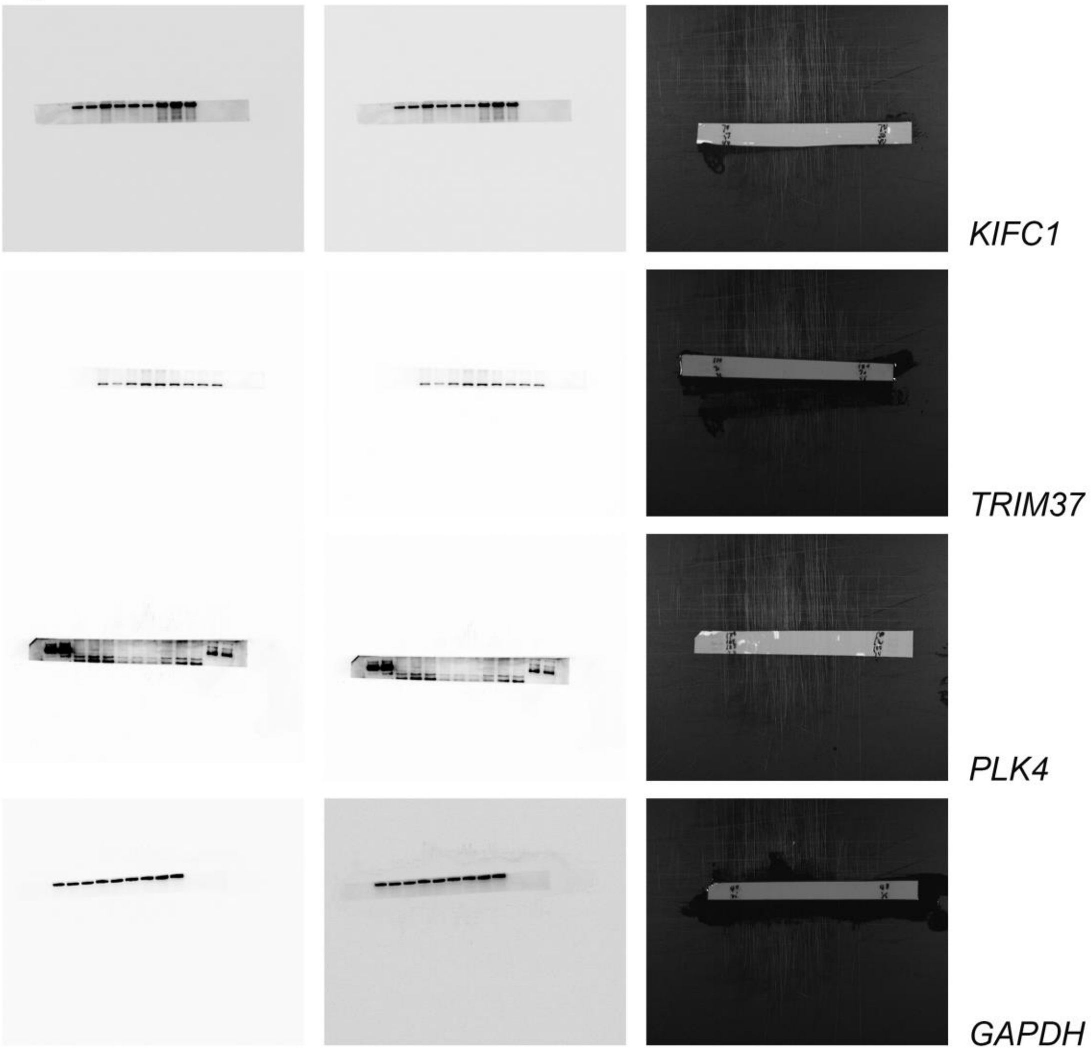

Figure S1 D

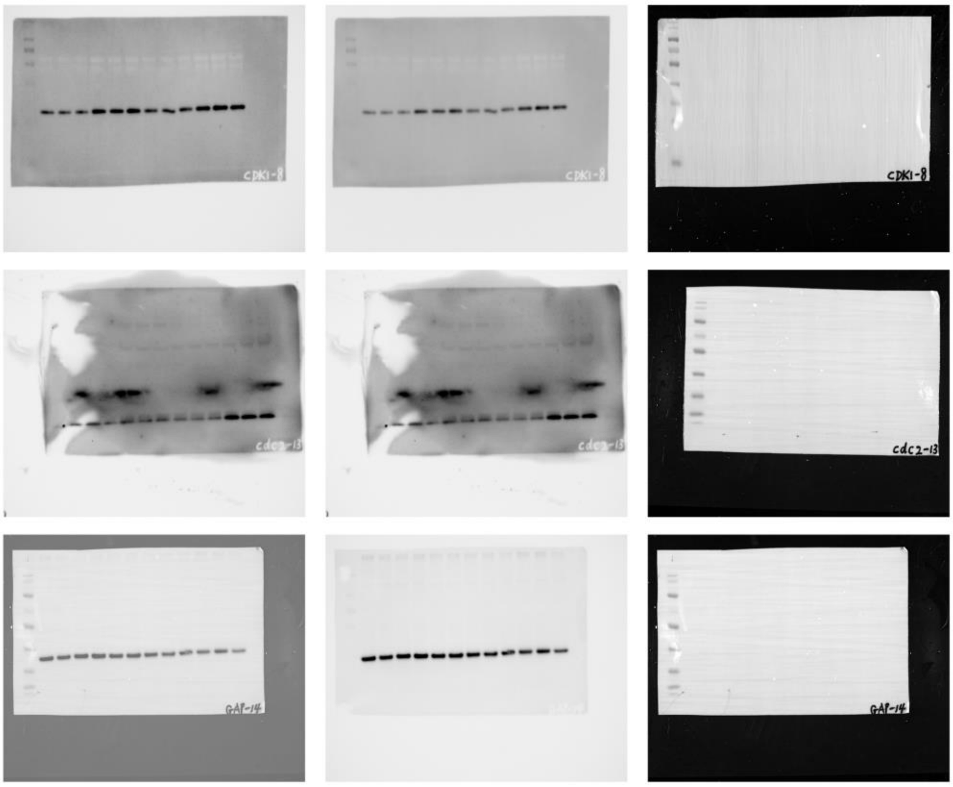

CDK1

CDC2

GAPDH

Figure S1 F

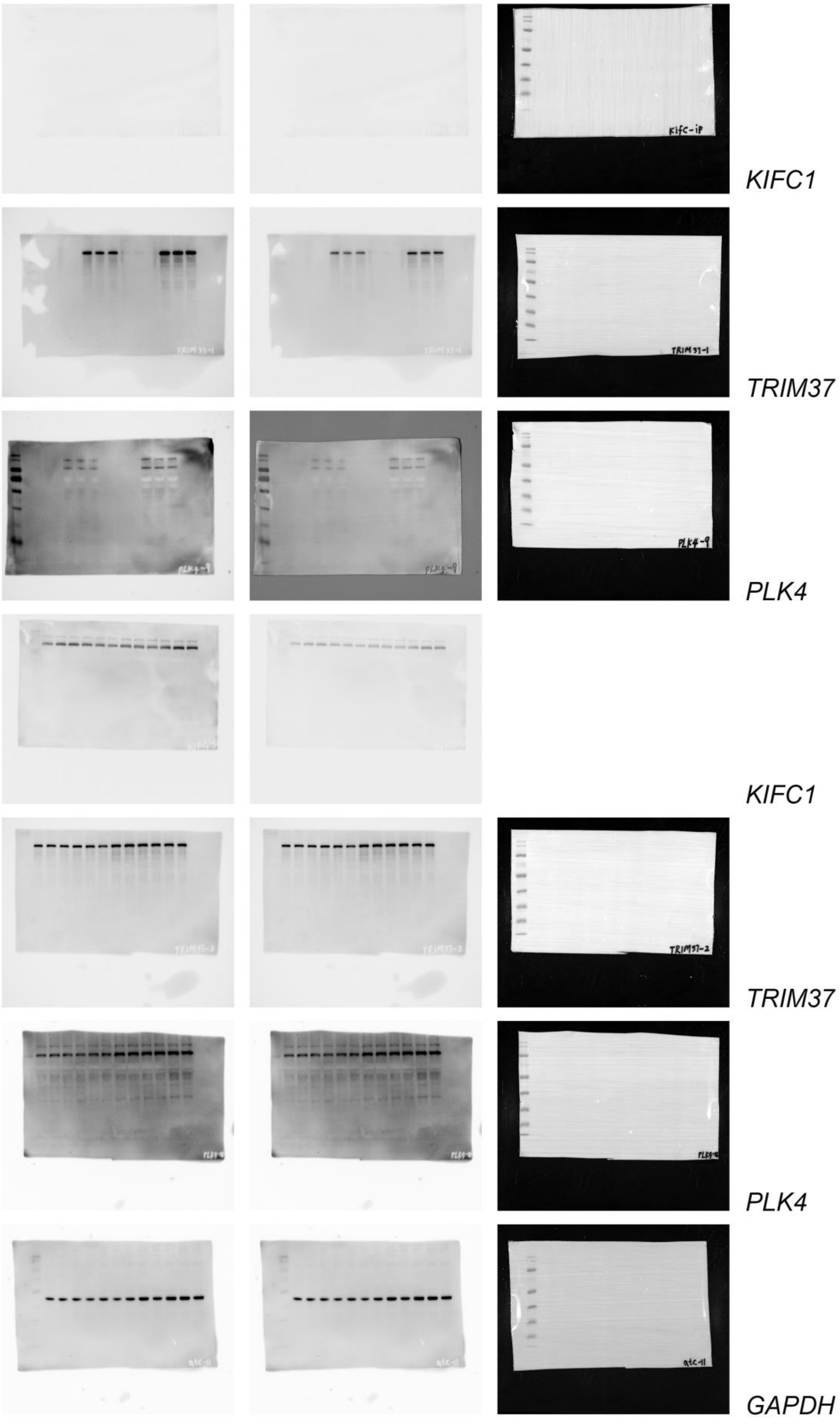

Figure S1 G

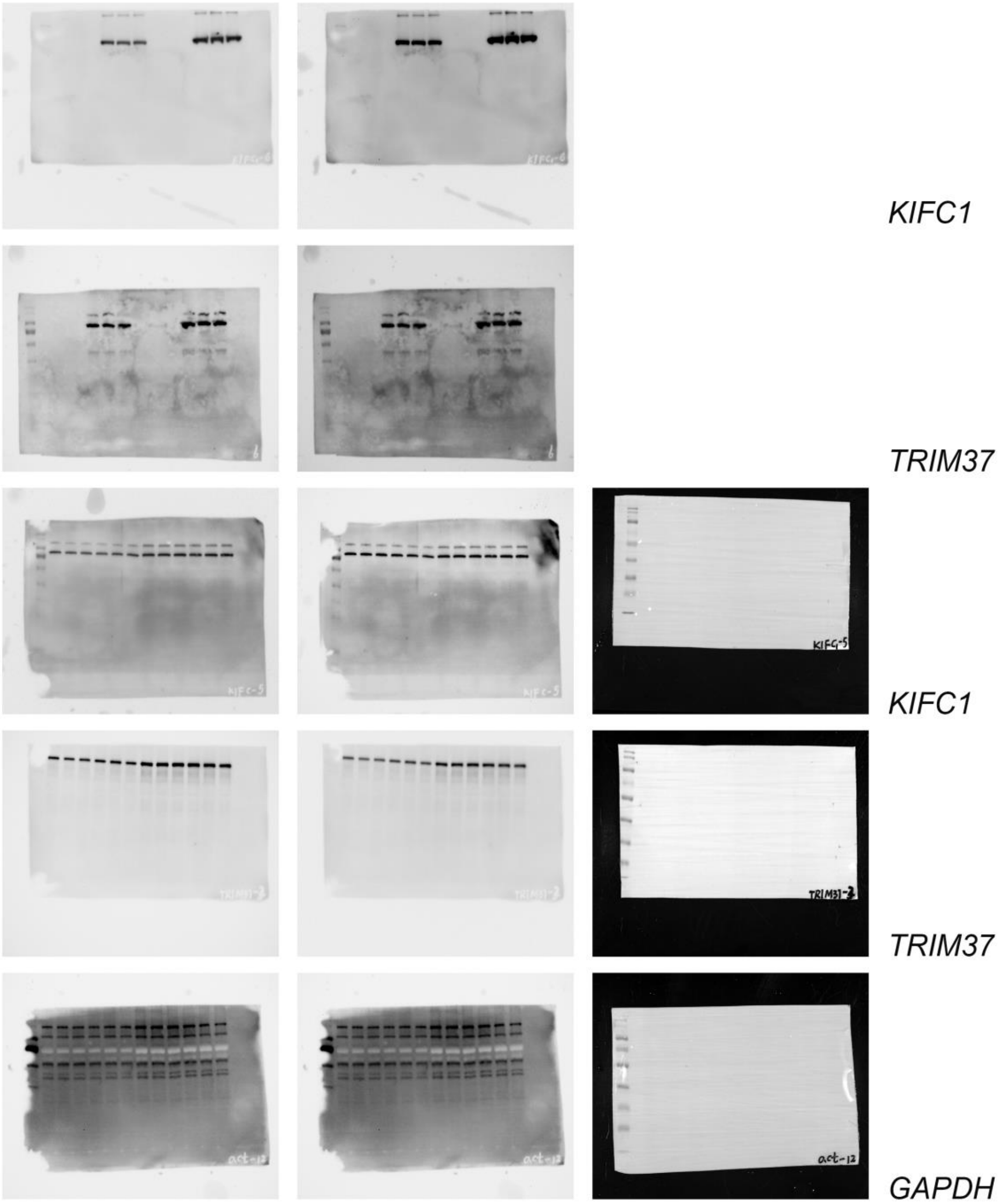

Supplement: Supplementary file 3 — original wb [file 41420_2024_2190_MOESM3_ESM.pdf]
